# A mutation in the *PRKAR1B* gene drives pathological mechanisms of neurodegeneration across species

**DOI:** 10.1093/brain/awae154

**Published:** 2024-05-14

**Authors:** Tal Benjamin-Zukerman, Gilat Shimon, Marie E Gaine, Anwar Dakwar, Netta Peled, Mohammad Aboraya, Ashar Masri-Ismail, Rania Safadi-Safa, Meir Solomon, Varda Lev-Ram, Robert A Rissman, Johanna E Mayrhofer, Andrea Raffeiner, Merel O Mol, Benney M R Argue, Shaylah McCool, Binh Doan, John van Swieten, Eduard Stefan, Ted Abel, Ronit Ilouz

**Affiliations:** The Azrieli Faculty of Medicine, Bar Ilan University, Safed 1311502, Israel; The Azrieli Faculty of Medicine, Bar Ilan University, Safed 1311502, Israel; Department of Pharmaceutical Sciences and Experimental Therapeutics, University of Iowa, Iowa City, IA 52242, USA; Iowa Neuroscience Institute, University of Iowa, Iowa City, IA 52242, USA; The Azrieli Faculty of Medicine, Bar Ilan University, Safed 1311502, Israel; The Azrieli Faculty of Medicine, Bar Ilan University, Safed 1311502, Israel; The Azrieli Faculty of Medicine, Bar Ilan University, Safed 1311502, Israel; The Azrieli Faculty of Medicine, Bar Ilan University, Safed 1311502, Israel; The Azrieli Faculty of Medicine, Bar Ilan University, Safed 1311502, Israel; The Azrieli Faculty of Medicine, Bar Ilan University, Safed 1311502, Israel; Department of Pharmacology, University of California, San Diego, La Jolla, CA 92093, USA; Department of Physiology and Neurosciences, Alzheimer’s Therapeutic Research Institute, Keck School of Medicine of University of Southern California, San Diego, CA 92121, USA; Institute of Molecular Biology, Institute of Biochemistry and Center for Molecular Biosciences, University of Innsbruck, Innsbruck, Tyrol 6020, Austria; Tyrolean Cancer Research Institute (TKFI), Innsbruck, Tyrol 6020, Austria; Institute of Molecular Biology, Institute of Biochemistry and Center for Molecular Biosciences, University of Innsbruck, Innsbruck, Tyrol 6020, Austria; Tyrolean Cancer Research Institute (TKFI), Innsbruck, Tyrol 6020, Austria; Department of Neurology and Alzheimer Center Erasmus MC, Erasmus University Medical Center, Rotterdam 3015 CE, The Netherlands; Department of Pharmaceutical Sciences and Experimental Therapeutics, University of Iowa, Iowa City, IA 52242, USA; Department of Pharmaceutical Sciences and Experimental Therapeutics, University of Iowa, Iowa City, IA 52242, USA; Department of Pharmaceutical Sciences and Experimental Therapeutics, University of Iowa, Iowa City, IA 52242, USA; Department of Neurology and Alzheimer Center Erasmus MC, Erasmus University Medical Center, Rotterdam 3015 CE, The Netherlands; Institute of Molecular Biology, Institute of Biochemistry and Center for Molecular Biosciences, University of Innsbruck, Innsbruck, Tyrol 6020, Austria; Tyrolean Cancer Research Institute (TKFI), Innsbruck, Tyrol 6020, Austria; Iowa Neuroscience Institute, University of Iowa, Iowa City, IA 52242, USA; Department of Neuroscience and Pharmacology, University of Iowa Carver College of Medicine, Iowa City, IA 52242, USA; The Azrieli Faculty of Medicine, Bar Ilan University, Safed 1311502, Israel; The Leslie & Susan Goldschmied (Gonda) Multidisciplinary Brain Research Center, Bar-Ilan University, 5290002 Ramat-Gan, Israel

**Keywords:** protein kinase A (PKA), PRKAR1B, neurodegenerative diseases, NLPD-PKA disease, protein aggregation, neuronal loss

## Abstract

Protein kinase A (PKA) neuronal function is controlled by the interaction of a regulatory (R) subunit dimer with two catalytic subunits. Recently, the L50R variant in the gene encoding the RIβ subunit was identified in individuals with a novel neurodegenerative disease. However, the mechanisms driving the disease phenotype remained unknown.

In this study, we generated a mouse model carrying the RIβ-L50R mutation to replicate the human disease phenotype and study its progression with age. We examined post-mortem brains of affected individuals as well as live cell cultures. Employing biochemical assays, immunohistochemistry and behavioural assessments, we investigated the impact of the mutation on PKA complex assembly, protein aggregation and neuronal degeneration.

We reveal that RIβ is an aggregation-prone protein that progressively accumulates in wildtype and Alzheimer’s mouse models with age, while aggregation is accelerated in the RIβ-L50R mouse model. We define RIβ-L50R as a causal mutation driving an age-dependent behavioural and disease phenotype in human and mouse models. Mechanistically, this mutation disrupts RIβ dimerization, leading to aggregation of its monomers. Intriguingly, interaction with the catalytic subunit protects the RIβ-L50R from self-aggregating, in a dose-dependent manner. Furthermore, cAMP signaling induces RIβ-L50R aggregation.

The pathophysiological mechanism elucidated here for a newly recognized neurodegenerative disease, in which protein aggregation is the result of disrupted homodimerization, sheds light on a remarkably under-appreciated but potentially common mechanism across several neurodegenerative diseases.

## Teaser

The *PRKAR1B* gene encodes a regulatory subunit of protein kinase A. Benjamin-Zukerman *et al.* generate a mouse model harbouring a variant in *PRKAR1B* previously detected in patients with a rare neurodegenerative disease, and identify the molecular mechanisms driving the disease phenotype.

## Introduction

Cyclic AMP-dependent protein kinase (PKA) regulates numerous neuronal signalling pathways. PKA function is important for learning and memory and is involved in multiple forms of synaptic plasticity, including hippocampal long-term potentiation.^1,2^ Dysregulation of PKA signalling contributes to neurodevelopmental disorders and neurodegenerative diseases^3-5^

PKA function is highly controlled by macromolecular assembly localized at specific cellular sites. The holoenzyme consists of a regulatory (R) subunit dimer bound to two catalytic (C) subunits. Mammalian cells possess four regulatory subunits (RIα, RIβ, RIIα and RIIβ) encoded by different genes (*PRKAR1A*, *PRKAR1B*, *PRKAR2A* and *PRKAR2B*, respectively).^6^ The PKA holoenzyme structures revealed that the tetrameric assembly is isoform-specific and is dictated by the flexible linkers of the R-subunits that allow the formation of distinct structures.^7-10^ These quaternary holoenzyme structures (RIα_2_:C_2_, RIβ_2_:C_2_, RIIα_2_:C_2_ and RIIβ_2_:C_2_) differ in their allosteric mechanism and their sensitivity to cAMP.

All R subunits share the same domain organization, which includes a dimerization and docking (D/D) domain, followed by an inhibitor sequence and two cAMP binding domains. The D/D domain of each R-subunit forms an isologous dimer in which the same binding sets on the two subunits complete with one another when they are rotated 180° relative to one another.^11-13^ Such interaction between the two R-subunits creates a hydrophobic groove that provides a docking surface for an A-kinase anchoring protein (AKAP) that binds through an amphipathic helix; the hallmark signature motif of all AKAPs.^14,15^ The assembly of PKA holoenzymes with various AKAPs target the complex at a specific intracellular microdomain and provides additional specificity to PKA function.^16-20^

Many disease-associated mutations in R-subunits have been linked to changes in protein expression levels and consequently to altered kinase function.^21-23^ Several point mutations have been shown to alter cAMP binding affinity or the direct or indirect release of C-subunits.^24-28^ So far, all identified patient mutations do not interfere with the structural assembly of the holoenzymes, which has been extensively studied *in vitro.*^8,10^

Recently, the c.149T>G (p.Leu50Arg) missense mutation was identified in individuals diagnosed with a rare neurodegenerative disease characterized by dementia and/or parkinsonism. The pathogenic mutation is in *PRKAR1B*, coding for the RIβ subunit, and the protein produced has been found in aggregates in various brain regions of affected individuals.^4^ In this study, we report an additional patient carrying this mutation and we propose the name neuronal loss and parkinsonism driven by a PKA mutation (NLPD-PKA), which reflects the key pathological features observed in individuals with this disease. Indeed, the RIβ subunit is highly expressed in the brain and exhibits a distinct and unique pattern of expression in various brain regions when compared to RIIβ.^29,30^ Various studies in mice demonstrated that one isoform cannot compensate for the absence of another.^31,32^

In this study, we established the role of the L50R mutation as a driver of disease by creating a mouse model carrying the mutation. We found that while altered motor performance manifested at a later stage, similar to affected individuals, protein aggregation and loss of Purkinje cells are accelerated in young RIβ-L50R heterozygous mice. The pathological effects are driven by structural defects in the assembly of the PKA holoenzyme and its allosteric regulation which are conserved between humans and mice.

## Materials and methods

Additional materials and methods are available in the Supplementary material.

### Subjects

Human brain tissues were obtained from the Netherlands Brain Bank. The study was approved by the medical ethical committee of the Erasmus Medical Center Rotterdam, and all family members participating in the study, or their legal representatives gave informed consent. The sex, age at death and clinical diagnosis of the study individuals are summarized previously.^4^ Individuals III:2 and III:4 were used in the study. Control subjects did not show neurodegenerative changes at pathological examination.

### Mouse model

The Genome Editing Facility at the University of Iowa used the clustered regularly interspaced short palindromic repeats (CRISPR)/Cas9 nuclease system to introduce the L50R mutation into mice. Template DNA with a three-base mutation (TCC/GGA) was used to replicate the L50R mutation and amino acid change seen in individuals.^4^ Two additional silent mutations were included to inhibit the guide RNA from rebinding to previously cut DNA. The mutation was confirmed using Sanger sequencing [Supplementary Fig. 2A(ii)]. Mice were housed in same-sex groups of up to five under 12-h light/12-h dark cycle with *ad libitum* access to food and water in a temperature- and humidity-controlled room. All experiments were conducted according to the National Institutes of Health guidelines for animal care and were approved by the Institutional Animal Care and Use Committee at the University of Iowa.

### Cultured cell lines

Rat pheochromocytoma (PC12) or human embryonic kidney (HEK)293T cells were grown in Dulbecco’s modified Eagle medium (DMEM) high glucose medium (Biological Industries), supplemented with 10% fetal bovine serum (Biological Industries), 2 mM GlutaMAX (GIBCO) and 5% penicillin-streptomycin (Biological Industries). Proliferating cell cultures were maintained in a 37°C incubator with 5% CO_2_. Cells were tested for mycoplasma every 3 months using a Mycoplasma PCR Kit (Hylabs, Cat. No. KI50341).

## Results

### Clinical and pathological features of individuals heterozygous for the RIβ-L50R mutation

**Figure 1:**
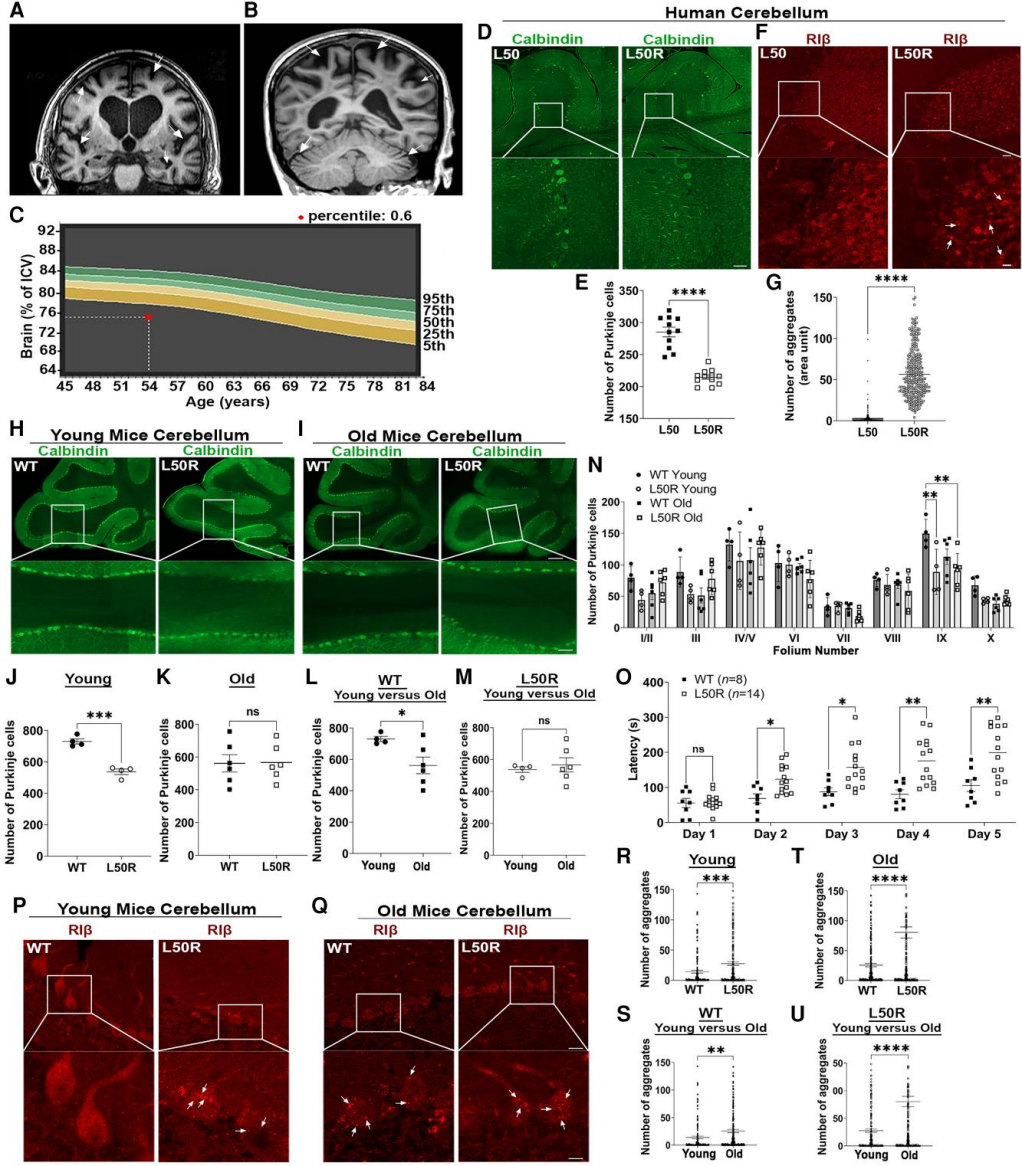
**The RIβ-L50R mouse model recapitulates neuronal loss, protein aggregation and exhibits behavioural changes**. (**A**) Coronal MRI image of a previously described individual heterozygous for the RIβ-L50R mutation.^4^ (**B**) Sagittal MRI image from a newly described individual with RIβ-L50R mutation. Arrows point to regions with atrophy in the cortex and cerebellum. (**C**) Quantification of brain atrophy as defined by percentage of intracranial volume (ICV) plotted against the reference centile curves in a normal population. The red dot represents the ICV of the new individual compared to the normal volume. (**D**) Human cerebellar tissue of a healthy individual (L50) and an L50R individual were immuno-stained with anti-calbindin antibody. Images acquired at ×20 magnification were stitched to form a larger picture. The white box represents the area from which subfield full-resolution images were captured. Scale bar = 200 µm; for enlarged image = 50 µm. (**E**) Quantification analysis of number of Purkinje cells. Each dot represents the total number of Purkinje cells in folia. (**F**) Cerebellum slice from a healthy individual (L50) and a patient heterozygous for the L50R generating mutation (L50R) were immuno-stained with anti-RIβ antibody. Protein aggregations are denoted by white arrows. Scale bar = 50 µm; for enlarged image = 20 µm. (**G**) Numbers of aggregates per unit were taken from three slices (100 dots per slice). Error bars represent ±standard error of the mean (SEM). Unpaired *t*-test. ****P < 0.0001. (**H** and **I**) Cerebellum slices from wild-type (WT) or L50R young mice (3–4 months old) (**H**) or old mice (16–20 months old) (**I**) immuno-stained with anti-calbindin antibody. Scale bar = 200 µm; enlarged image of folium number IX, scale bar = 50 µm. (**J**–**M**) Paired comparison analyses for number of Purkinje cells in a whole mouse folia for WT/RIβ-L50R and/or young/old mice as denoted. Each dot represents the number in a different mouse. (**N**) Quantitative analysis of total number of Purkinje cells in each individual lobule of mice cerebella. Two-way ANOVA with Tukey’s *post hoc* test. (**O**) Motor performance changes measured using the accelerating rotarod test for WT (*n* = 8) and L50R (*n* = 14) old mice (>16 months old). Three tests were performed per day for 5 days. Each dot represents one mouse. Error bars represent SEM. Repeated measures ANOVA with Šídák correction for multiple testing. *P < 0.05, **P < 0.01, ***P < 0.001, ns = non-significant comparison. (**P** and **Q**) Cerebellum slices from WT or L50R young mice (3–4 months old) (**P**) or old mice (16–18 months old) (**Q**) immuno-stained with anti-RIβ antibody. (**R**–**U**) Image quantification of RIβ protein aggregation per Purkinje cell of either WT and/or L50R young mice (3–4 months old) and/or old mice (16–18 months old). Paired comparison analyses performed as denoted in each graph. Each dot represents the number of aggregates per cell. Error bars represent ±SEM. Unpaired *t*-test. **P < 0.01, ***P < 0.001, ****P < 0.0001.

We report a new case involving a 57-year-old female who carries the L50R heterozygous mutation in the *PRKAR1B* gene. The clinical characteristics of this individual closely resemble those of previously reported patients.^4^ Symptoms began to manifest at the age of 54, with the onset age varying among affected individuals, ranging from 45 to 64 years old. Upon evaluation, the patient exhibited short-term memory deficits and lack of planning skills. Scoring 23 on the Mini-Mental State Examination and 13 on the Frontal Assessment Battery. At neurological examination, a mild tremor of both arms with mild rigidity was observed in addition to a slow and unstable gait with a forward lean. It was also noted that finger-to-nose test was slow and unsteady. MRI of two patients with the mutation revealed severe global cortical atrophy, most prominent in parietal and frontal areas, and relatively spared in occipital and temporal lobes. Cerebellum examination revealed slight atrophy (Fig. 1A and B). Quantitative assessment of brain MRI, performed as previously described,^33^ revealed abnormal brain volume with more progressive decline than the reference centile curve (Fig. 1C and Supplementary Fig. 1).

The motor performance changes and cerebellar atrophy led us to analyse neuronal loss by immunohistochemical (IHC) analysis on human cerebellum slices using calbindin, a specific marker for Purkinje cells. The patient with the RIβ L50R mutation displayed a significant loss of Purkinje cells at the age of 61 compared to the healthy individual (Fig. 1D and E). To analyse the cerebellum at the subcellular level, we performed IHC assays on human cerebellum sections with RIβ-specific antibodies. We found diffuse cytoplasmic staining of RIβ in a healthy individual, unlike the RIβ protein aggregates in the individual with the L50R mutation (Fig. 1F and G), consistent with the previous report.^4^

### Mutant RIβ-L50R drives neuronal loss, altered motor performance and protein aggregation in a mouse model

To elucidate whether the L50R mutation in RIβ is an evolutionarily conserved mutation that drives the neuropathology of the disease and whether it plays a direct role in protein aggregation, we generated a heterozygous mouse model. The L50R mutation was introduced via CRISPR/Cas9 genome editing technology [illustrated in Supplementary Fig. 2A(i–iii)]. No off-target effects were generated following the introduction of the specific mutation [Supplementary Fig. 2B(i–iii)]. We first analysed the mouse brains at the cellular level. Consistent with previous reports, we observed a significant age-related loss of Purkinje cells in aged wild-type (WT) mice (Fig. 1L).^34^ This neuronal loss is accelerated in mice carrying the mutation and is prominent at a young age (Fig. 1H–M), similar to what we found in individuals with the same mutation. The neuronal loss is specifically accelerated at folium IX (Fig. 1N).

We initially investigated motor performance using the accelerating rotarod paradigm to examine whether the mutation drives behavioural changes in the mouse model and correlate the changes with the human diagnosis described above. We performed three tests per day for 5 days to test motor performance. In old mice (>16 months old), a difference was noted starting on Day 2. The mice with the mutation show a significantly increased latency compared to the WT mice despite marked neuronal loss (Fig. 1O). This change was evident in old mice but not in young mice (Supplementary Fig. 3A). We compared weight to age in both WT and RIβ-L50R mutant mice and no difference was noted, confirming that the decreased latency is not due to increased body weight. Using activity monitoring, we found that our L50R mouse model displayed hyperactivity, and this was more predominant in the dark (active) phase of the diurnal cycle (Supplementary Fig. 3B). A correlation between increased rotarod performance and hyperactivity has previously been noted in other neurological mouse models.^35-37^ Notably, this enhancement would be consistent with the cerebellar deficits also seen in the L50R mouse model. Disruption of the cerebellum has been found in several neurodevelopmental disorders, including autism spectrum disorder (ASD) and attention deficit-hyperactivity disorder.^38^ GSK3b overexpression in cerebellar Bergmann glia cells has been associated with improved rotarod performance^39^ and hyperactivity has been seen in mice with cerebellar damage.^40^ Overall, our L50R mouse model has a significant age-dependent phenotype, that we hypothesize represents altered motor performance and hyperactivity caused by cerebellar dysfunction.

To examine whether the mutation leads to learning and memory deficits as seen in individuals, we used the Barnes maze paradigm and fear conditioning. The results suggest that the mutation does not lead to memory deficits in mice (Supplementary Fig. 3C and D). Additionally, we examined anxiety by using the elevated zero maze test, and these results suggested that the mutation does not cause anxiety (Supplementary Fig. 3E). Overall, we present a new mouse model that recapitulates accelerated loss of Purkinje cells and alternation in motor performance and hyperactivity.

To investigate whether L50R is a driver mutation for RIβ protein aggregation and how the aggregation progresses with age, we examined RIβ protein aggregation in mice at a young age (3–4 months old) and an old age (16–20 months old) in various brain regions using a specific RIβ antibody in IHC assays. It is evident from a young age that the mutant mice exhibit a significant increase in RIβ protein aggregation in the cerebellar Purkinje cells compared to their WT littermates (Fig. 1P and R). The number of RIβ aggregates in these cells is significantly increased in old WT mice, suggesting that RIβ is prone to aggregation (Fig. 1Q and S). The mutation, however, accelerates this process as protein aggregation occurs earlier than WT, and the number of protein aggregates in the mutant is significantly higher than in WT at both young and old ages (Fig. 1R–T). To investigate RIβ protein aggregation, we used the well-established double transgenic PSAPP mouse model, which is commonly associated with Alzheimer’s disease. Interestingly, only age-related accumulation of RIβ aggregates was observed in these mice, suggesting that the acceleration of aggregation is specific to the RIβ-L50R mice (Supplementary Fig. 4).

The quantitative analyses performed on the L50R mouse brains provide consistent results with the human brain, providing evidence that these accumulations begin earlier and increase with age (Fig. 1U).

### RIβ-L50R mutant disrupts the R subunit dimer interface, leading to monomer aggregation across species

**Figure 2:**
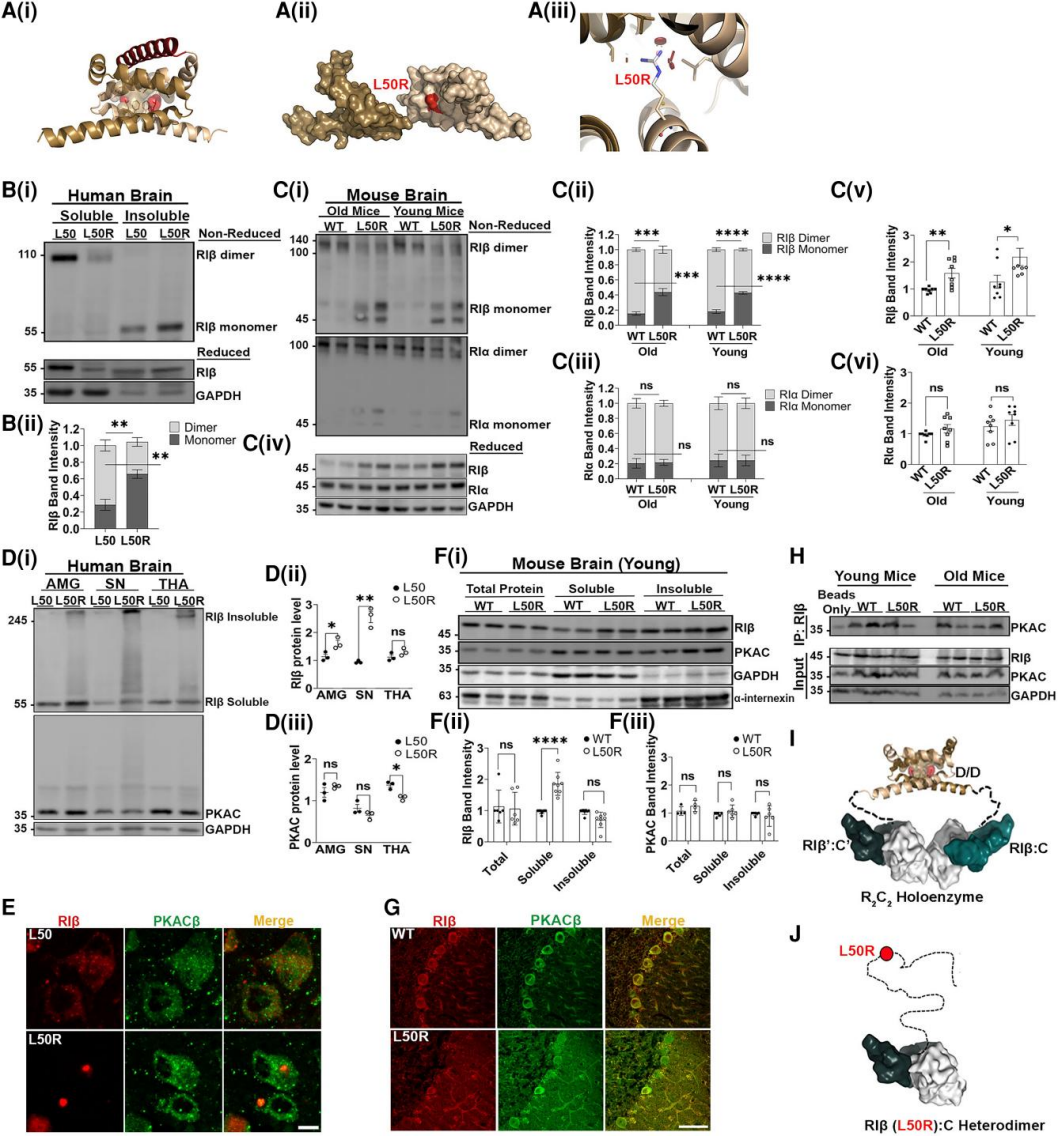
**The RIβ-L50R mutant disrupts RIβ dimerization and prevents PKA holoenzyme assembly**. [**A**(**i**)] Overall structure of the RIβ dimerization and docking (D/D) domain. One promoter is coloured brown and the other sand. The hydrophobic groove created by the two protomers serves as a docking site for an A-kinase anchoring protein (AKAP) peptide (red). The position of residue L50 (red spheres) in both protomers and the surrounding residues (brown spheres) are shown. [**A**(**ii**)] The position of L50R is shown as a red molecular surface between the two protomers. [**A**(**iii**)] *In silico* mutagenesis of the RIβ D/D domain structure (Protein Data Bank: F9K), introducing Arg instead of Leu at position 50 using PyMOL software. The model exhibits clashes with surrounding residues from the other protomer (red discs). [**B**(**i**)] Flash-frozen human cerebellum tissue lysates from a healthy individual (L50) and an individual heterozygous for the RIβ-L50R-generating mutation (L50R) were separated by sodium dodecyl-sulfate polyacrylamide gel electrophoresis (SDS-PAGE) under non-reduced (*top*) and reduced (*bottom*) conditions. In the L50 patient, RIβ is enriched in the cytosol and expressed as a homodimer, which is stabilized by inter-disulfide bonds. In the L50R patient, RIβ is found in the insoluble fraction and migrates as a monomer, confirming the breaking of dimerization. SDS-PAGE under reduced conditions compares total expression levels of RIβ between L50 and L50R in enriched fractions. GAPDH was used to determine equal loading as well as the quality of subcellular fractionation. Patient IDs (2012-95/S12/095). [**B**(**ii**)] RIβ band intensity was quantified from three independent experiments to identify the ratio between RIβ monomers to dimers. [**C**(**i**)] Tissues of soluble lysates from old (16–20 months) or young (3–4 months) of wild-type (WT) or L50R heterozygous littermate mice were separated by SDS-PAGE under non-reduced and membrane was exposed to RIβ specific antibody (*top*) or RIα specific antibody (*bottom*). [**C**(**ii**)] RIβ monomers/dimers band intensity quantifications from three independent experiments. [**C**(**iii**)] RIα monomers/dimers band intensity quantifications from three independent experiments. [**C**(**iv**)] Proteins from soluble lysates were separated by SDS-PAGE under reduced conditions to compare total expression levels of RIβ and RIa in the soluble fractions and the relative dimer formation in the non-reduced gel. GAPDH was used to determine equal loading. Band intensity for RIβ and RIa is quantified in **C**(**v**) and **C**(**vi**), respectively. Each dot represents a different mouse from at least three independent experiments. [**D**(**i**)] Flash-frozen human amygdala (AMG), substantia nigra (SN) and thalamus (THA) tissue lysates from a healthy individual (L50) and a patient carry the L50R mutation (L50R) were separated by SDS-PAGE under reduced conditions. In the L50R patient, RIβ migrates at its expected molecular weight (55 kDa) as well as in higher-order assemblies. The catalytic (C)-subunit is not detected in at higher-order assemblies and shows similar protein expression levels in both L50 and L50R patients. GAPDH was used to determine equal loading. [**D**(**ii**)] Quantification of RIβ band intensity (sum of monomer and multimer forms) from three independent experiments. Error bars represent ±standard error of the mean (SEM). Unpaired *t*-test. *P < 0.05, **P < 0.01; ns = non-significant comparison. [**D**(**iii**)] Quantification of C-subunit band intensity from three independent experiments. Unpaired *t*-test. (**E**) Paraffin-embedded human tissues from thalamus of L50 and L50R. The tissues were immuno-stained with anti-RIβ (red) and anti-PKACβ (PRKACB; green) antibodies. The images were acquired using confocal microscopy with ×63 magnification. Scale bar = 10 µm. [**F**(**i**)] Total cell lysates of cerebellum from WT and RIβ-L50R mutant mice at 3–4 months old were extracted and separated to soluble and insoluble fractions. Proteins were loaded on SDS-PAGE and probed with indicated antibodies. GAPDH and α-internexin were used as loading controls for soluble and insoluble fractions, respectively. [**F**(**ii** and **iii**)] Quantifications of band intensity of RIβ (**ii**) or PKAC (**iii**). Total proteins and soluble fractions were normalized to GAPDH and insoluble fractions were normalized to a-internexin. Each dot represents lysates from a different mouse. Error bars represent ±SEM. Unpaired *t*-test. **P < 0.01, ****P < 0.0001; ns; non-significant comparison. (**G**) IHC staining of cerebellum sagittal sections from WT and RIβ-L50R mutant young mice at 3–4 months old. The sections were labeled with anti-RIβ (red) and anti-PKACβ (green) antibodies. Images were obtained with confocal microscopy at ×63 magnification. Scale bar = 10 µm. (**H**) Immunoprecipitation assay using mice cerebellum lysates at both 3–4 and 16–20 months old. Lysates were immunoprecipitated with anti-RIβ antibody. Samples were loaded on SDS-PAGE and probed with anti-PKAC antibody. Lower panels: expression levels of RIβ, PKAC and GAPDH were detected in total cell lysates (Input) using specific antibodies. (**I**) Schematic representation of the RIβ holoenzyme structure showing how the D/D domain is extended away from the two R:C heterodimers by a flexible linker. The quaternary structure allows assemblies of RIβ:RIβ homodimerization and RIβ:C dimers of heterodimers. (**J**) Depiction of the RIβ-L50R mutant which retains the capacity to bind to the C-subunit but is unable to dimerize. The R-subunit is in teal, and the C-subunit is in white. PKAC = cAMP-dependent protein kinase catalytic subunit.

To understand the effect of the mutation leading to the L50R replacement on the RIβ holoenzyme structure, we analysed the L50 position within the RIβ D/D domain structure and its surroundings. RIβ dimerization occurs upon interlocking of two monomers into a 2-fold symmetrical structure that comprises three α-helices [Fig. 2A(i)]. Each monomer contains hydrophobic and polar residues that stabilize the dimerization. The L50 residue is located at the dimer interface of the two protomers and is critical for packing and stabilizing the hydrophobic core residues [Fig. 2A(ii)]. We predicted that introducing a bulky positively charged Arg instead of the small non-polar Leu at this position would perturb the dimer formation. *In silico* analysis predicted that this amino acid substitution would cause steric clashes with the surrounding residues and thus cannot be accommodated [Fig. 2A(iii)]. The L50 residue is highly conserved across eukaryotes, and we identified more aggregation-prone segments in the D/D domain sequence of humans than in other species (Supplementary Fig. 5A, red boxes).

To validate the *in silico* modelling biochemically, we extracted cell lysates from the cerebellum taken from a healthy individual (denoted as L50 in Fig. 2B) and an individual who was identified as heterozygous for the L50R mutation (Fig. 2B). We isolated the soluble protein pool to enrich the population of the R subunit dimers and/or monomers. Lysates were loaded and separated by sodium dodecyl-sulfate polyacrylamide gel electrophoresis (SDS-PAGE) under reduced conditions to determine the total expression levels of RIβ as well as under non-reduced conditions, to analyse dimerization formation between the two protomers as these are stabilized by inter-disulfide bonds. Consistent with the *in silico* structural prediction, the RIβ dimer was enriched in the soluble protein fraction from a healthy individual, whereas only a faint band corresponding to the R subunit dimer was detected in the L50R heterozygous patient [Fig. 2B(i)]. In this patient, RIβ proteins were enriched in the SDS-containing fraction and were detected as monomers when SDS-PAGE was performed under oxidizing conditions [Fig. 2B(i)]. The ratio between monomer and dimers was quantified [Fig. 2B(ii)].

To identify whether the heterozygous L50R mutation interferes with dimer formation across species, we extracted cell lysates from mice to perform biochemical assays to analyse dimer formation as detected in the human samples. Soluble fractions of brain lysates from young and old mice were separated on SDS-PAGE under non-reduced conditions. Consistent with the human brain lysate, the RIβ proteins of the WT mice form dimers, whereas only 50% of the proteins exist as dimers in the heterozygous mice [Fig. 2C(i), top]. The ratio between dimers and monomers was quantified [Fig. 2C(ii)]. We then exposed the same membrane to the RIα-specific antibody as a control for dimer formation. The RIα proteins were detected mostly as dimers at equal amounts in all samples suggesting that the L50R mutation interferes only with RIβ homodimer formation [Fig. 2C(i, bottom, and iii)]. The same samples were loaded on SDS-PAGE under reducing conditions to determine the total expression levels of the detected proteins [Fig. 2C(iv)]. Band intensities for RIβ and RIα proteins from at least three different experiments were quantified and are shown [Fig. 2C(v and vi)].

### Catalytic subunit expression is not affected by the L50R mutant in human and mouse brain

To evaluate expression levels of RIβ and the C-subunit, we performed western blot analysis on total cell lysates extracted from healthy individuals and patients that carry the mutation, after SDS-PAGE was performed under reducing conditions. Immunoblotting with an antibody against RIβ showed that patients expressing the L50R mutant had significantly higher expression levels of RIβ-L50R compared to healthy individuals (L50) in both the amygdala and substantia nigra [Fig. 2D(i and ii)]. The expression levels of the C-subunit showed no significant change in healthy and affected individuals [Fig. 2D(i and iii)]. Additionally, these gels revealed higher-order assemblies of RIβ-L50R but not of the C-subunit, as the latter was observed at its expected molecular weight in brain lysates extracted from amygdala, substantia nigra and thalamus [Fig. 2D(i)]. When the same brain regions were analysed by IHC to assess protein distribution and aggregation, round and compact cytoplasmic inclusions with strong immune-reactivity for RIβ were seen in each of these brain regions in patients expressing the L50R mutant. These inclusions were not, however, immuno-reactive for the PKA C-subunit (PKAC). Instead, PKAC in L50R patients was diffusely distributed throughout the cytoplasm, as observed in healthy individuals (Fig. 2E and Supplementary Fig. 5B–E). Taken together, these biochemical assays and microscopy studies of several brain regions consistently suggest that RIβ-L50R aggregates without the C-subunit, which remains diffuse in the cytoplasm.

To compare the protein expression levels as well as the localization of RIβ and the C-subunit, we extracted lysates from the cerebellum of both WT and mutant mice at young and old ages. The data presented in Fig. 2F(i–iii) indicates that the mutation did not affect the total expression level of RIβ or the C-subunit in the mouse model. This is consistent with the RNA data presented in Supplementary Fig. 2. However, when total proteins were separated into soluble and insoluble fractions, we detected a significant increase in the level of soluble RIβ-L50R proteins compared to the level of soluble RIβ-WT proteins. The mutation did not have an effect on the C-subunit proteins in the same fraction. GAPDH and a-internexin were used to determine equal loading and to ensure the quality of soluble and insoluble subcellular fractionation, respectively. The band intensities of RIβ and were quantified from three independent experiments for each fraction [Fig. 2F(ii and iii)]. Similar patterns of expression in total cell lysates, soluble and insoluble fractions were detected at an old age (Supplementary Fig. 6B–D), suggesting that the subcellular changes driven by the mutation occur at a young age.

To investigate the differential expression pattern of RIβ and PKAC in the cerebellum of both WT and RIβ-L50R mice at young and old ages, we stained cerebellum slices by IHC with specific antibodies. We found that in the WT young mice, RIβ and PKAC were diffused in the cytoplasm and co-localized (as shown in Fig. 2G for ×63 magnification and Supplementary Fig. 6A for ×20 magnification). In the RIβ-L50R mouse model at both young and old ages, the RIβ-L50R proteins were localized in cytoplasmic aggregates, while the expression pattern of PKAC remained diffuse in the cytoplasm suggesting that the latter protein is not affected by the presence of the mutation (Fig. 2G and Supplementary Fig. 6). Interestingly, in the PSAPP mouse model with the age-dependent accumulation of RIβ, the expression pattern of PKAC was not altered, indicating that the changes were specific to the RIβ in both models (Supplementary Fig. 4). To detect whether PKAC interacts with RIβ in the presence of the L50R mutation in both young and old mice, we performed an immunoprecipitation (IP) assay using mouse cerebellum lysates. Lysates were immunoprecipitated with an RIβ-specific antibody, and samples were loaded on SDS-PAGE and probed with PKAC-specific antibody. The results presented in Fig. 2H confirm that PKAC interacts with RIβ and RIβ-L50R proteins in samples from both young and old mice. Before performing the IP assay, we loaded total cell lysates (Input in Fig. 2H) on SDS-PAGE and probed them with specific RIβ, PKAC and GAPDH antibodies to ensure similar expression levels of the detected proteins in all samples (Fig. 2H).

In summary, the PKA RIβ-specific holoenzyme comprises a dimer of two heterodimers, each composed of RIβ and C subunits. RIβ: RIβ homodimers bind to two C-subunits to form two (RIβ:C) heterodimers (Fig. 2I). The structural analysis of the RIβ holoenzyme assembly together with the biochemical assays on both human and mouse samples thus supports the findings that the mutation resulting in RIβ-L50R disrupts RIβ homodimerization but allows interactions with the PKA C-subunit (Fig. 2J).

### Quantification of RIβ-L50R PKA holoenzyme macromolecular assemblies in cells

**Figure 3:**
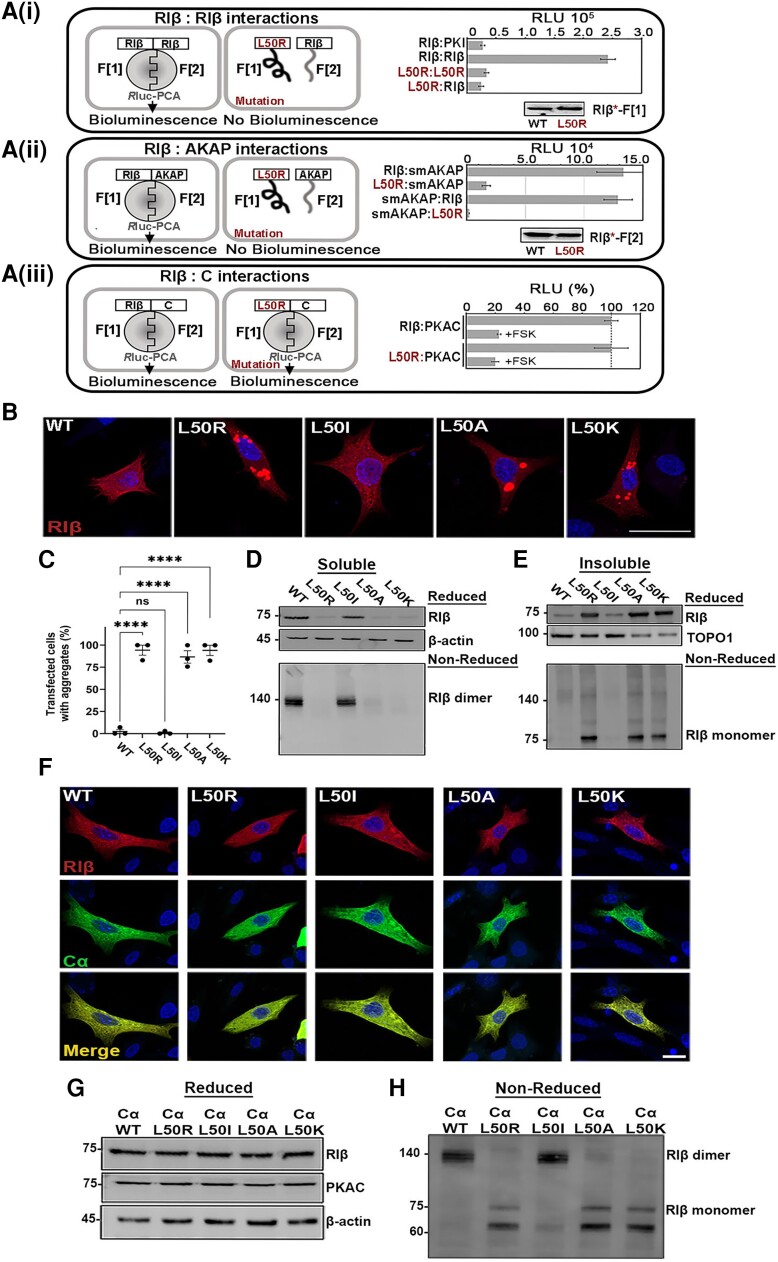
**The RIβ-L50R mutant protein disrupts RIβ:RIβ homo-dimerization, leading to protein aggregation in dissociated cells, but the formation of RIβ:C heterodimers protects against protein aggregation**. [**A**(**i**)] Schematic illustration of the *Renilla* luciferase (Rluc) protein-fragment complementation assay (PCA) biosensor strategy used to quantify protein-protein interactions of wild-type (WT) or L50R mutant RIβ hybrid proteins in human embryonic kidney (HEK)293 cells. The two RIβ monomers dimerize, as quantified by a bioluminescence readout, whereas the RIβ-L50R mutant in one or both protomers abolishes dimerization. PKI binding was used as a negative control. Immunoblot of the Rluc-F[1] fused to WT or mutant RIβ using a Rluc-F[1] specific antibodies confirms that the expression levels were the same. [**A**(**ii**)] *Left*: Schematic illustration of RIβ WT or the L50R mutant interacting with an A-kinase anchoring protein (AKAP) peptide in the *R*luc PCA system. *Right*: The RIβ-L50R mutant disrupts binding to smAKAP. Swapping the tags of the Rluc F[1] or F[2] that were C-terminally fused to RIβ or smAKAP had no effect. Immunoblot confirms that the expression levels of the proteins were the same. The figures are representative of three independent experiments (±standard deviation, technical experiments). [**A**(**iii**)] *Left*: Schematic illustration of RIβ-WT or the L50R mutant with the catalytic subunit (PKAC) in the *R*luc PCA. *Right*: The L50R mutation does not affect RIβ:PKAC complex formation or dissociation of the complex upon forskolin (FSK) treatment. (**B**) Confocal microscopy images of transiently transfected rat pheochromocytoma (PC12) cells expressing MKO2-tagged RIβ (WT or mutants). (**C**) The percentage of transfected cells that contains aggregates. Approximately 70–100 cells in total were counted for each transfection. Each dot represent an independent experiment. (**D** and **E**) Cell lysates of transiently transfected cells were divided into soluble (**D**) and insoluble (**E**) fractions, which were separated by sodium dodecyl-sulfate polyacrylamide gel electrophoresis (SDS-PAGE) under reduced conditions (*top*) or non-reduced conditions (*bottom*). β-Actin was used as a loading control for the soluble fraction. TOPO1 was used as a loading control for insoluble fraction. (**F**) Confocal images of PC12 cells co-transfected to express mKO2-tagged RIβ-WT or RIβ-mutants and mCerulean-Ca. Scale bar: 10 µm. (**G**) Soluble proteins of PC12 cell lysates were resolved by SDS-PAGE and immunoblotted with anti-RIβ or -Ca antibodies. β-Actin was used as a housekeeping protein loading control for each transfection. (**H**) The same protein samples as in **G** were separated by SDS-PAGE under non-reducing conditions and immunoblotted with anti-RIβ antibodies. The position of RIβ monomers and dimers are denoted.

To characterize and quantify direct interactions in living cells of PKA RIβ holoenzyme assemblies, we employed the *Renilla* luciferase (Rluc)-based protein-fragment complementation assay (PCA).^41^ Initially, we confirmed our structural and biochemical analysis by comparing homo- and heterodimerization of R*luc*-tagged RIβ WT and/or RIβ-L50R. The protein-protein interaction (PPI) signal decreased significantly upon RIβ-L50R substitution [Fig. 3A(i)]. This experiment demonstrated that an amino acid change in one of the RIβ protomers was sufficient to prevent RIβ homodimerization.

Next, we asked whether this mutation disrupted binding within the RIβ dimer to AKAPs. Since RIβ homodimerization is perturbed in the presence of the RIβ-L50R mutant, we expected reduced AKAP binding, given how its binding site normally presented by the regulatory subunit dimer was now eliminated. We measured binding by the R*luc*-tagged smAKAP, a peptide derived from an RI-specific AKAP known to bind RI subunits with high affinity.^18^ Indeed, using the PPI reporter, we observed a significant reduction in RIβ-L50R:smAKAP complex formation relative to native RIβ-based interactions [Fig. 3A(ii)].

Lastly, we explored whether the RIβ-L50R mutant could still bind the C-subunit to form the RIβ-L50R:C heterodimer. The mutation did not interfere with or affect the heterodimer formation nor its dissociation in response to forskolin (FSK) treatment [Fig. 3A(iii)]. The cellular and biochemical analyses of the RIβ holoenzyme assembly (Fig. 3A), together with the cell-based PPI assay, thus support the findings that mutation resulted in RIβ-L50R disrupts RIβ homodimerization and does not allow AKAP binding to the RIβ D/D domain but allows PKA C-subunit interactions (Fig. 3A).

### In dissociated cells, RIβ-L50R proteins do not dimerize and instead form insoluble aggregates

To assess whether the neuronal inclusions containing RIβ protein aggregation observed in human patient samples were directly caused by the L50R-encoding mutation and not an indirect result of an aberrant PKA signaling pathway driven by the mutation, we introduced the L50R encoding mutation into a mKO2 fluorescently tagged construct. We then transiently over-expressed the native (WT) or mutant RIβ in dissociated PC12 or HEK293T cells. However, as over-expression can lead to protein accumulation, which may lead to artefactual aggregation, we first characterized the L50K, L50I and L50A mutants as controls for over-expression-driven aggregation. Lys, like Arg, is a large positively charged residue; Ile structurally resembles Leu and should thus maintain interactions within the D/D domain like the native protein; whereas Ala is a small uncharged residue. As expected, cells transiently transfected with WT or the L50I R-subunit encoding constructs produced diffusely distributed RIβ protein in the cytoplasm of both PC12 and HEK293T cells, as revealed by IHC (Fig. 3B and C and Supplementary Fig. 7A and B).

To biochemically validate and analyse protein aggregation, we resolved soluble and insoluble protein fractions from PC12 or HEK293T cells. Separating these proteins on SDS-PAGE under reducing conditions revealed native (WT) and L50I RIβ as monomers in the soluble fractions. The same proteins, however, remained as dimers in SDS-PAGE performed under non-reducing conditions (Fig. 3D for PC12 cells and Supplementary Fig. 7C for HEK293T cells). The L50R disease-associated mutant, as well as the L50K and L50A mutants, were weakly detected in the soluble fraction but were readily found in the insoluble fractions in both cell lines (Fig. 3E for PC12 cells and Supplementary Fig. 7D for HEK293 cells). SDS-PAGE performed under non**-**reducing conditions did not reveal the L50R, L50K and L50A mutants at the position of dimers, unlike that seen with the WT and L50I proteins (Figs. 3D and E for PC12 and Supplementary Fig. 7C and D for HEK293 cells). All of the mutant proteins in the insoluble fractions appeared as aggregates in the IHC images (Fig. 3B and E for PC12 cells and Supplementary Fig. 7A and D for HEK293 cells). We thus concluded that having a positively charged residue such as Arg or Lys in place of L50 disrupts homodimerization, resulting in protein aggregation. We also found that Ala, a small uncharged residue, disrupted the dimerization, leading to RIβ aggregates (Fig. 3B and E for PC12 cells and Supplementary Fig. 7A and D for HEK293 cells). We suggest that the presence of a small uncharged residue at this position weakens dimer stability due to a loss of inter-subunit contacts, yielding protein aggregates. These results demonstrate that the disease-associated L50R mutant drives RIβ protein aggregation.

### The C-subunit protects RIβ***-***L50R from aggregation in the heterodimer RIβ-L50R:C complex

To analyse the interaction of PKAC with the mutant RIβ subunit and the resulting effect on aggregate formation, we co-expressed mKO2-tagged RIβ (WT or the L50R, L50I, L50A or L50K mutants) at similar protein levels as mCerulean-Cα in PC12 cells or HEK293T cells. Surprisingly, the co-expression resulted in diffuse localization of the heterodimer in the cytoplasm, despite the presence of the L50R mutant or other mutants that served as controls (Fig. 3F for PC12 cells and Supplementary Fig. 7E for HEK293T cells). Biochemical analyses of cell lysates by SDS-PAGE under reducing conditions validated that, similar to WT proteins, all mutant RIβ proteins were in the soluble fractions when the heterodimer complexes formed (Fig. 3G in PC12 cells and Supplementary Fig. 7F in HEK293T cells). As expected, the RIβ-L50R monomer, as well as the RIβ-L50R:Cα complex, could not homodimerize (Fig. 3H in PC12 and Supplementary Fig. 7G in HEK293T cells). These results, realized in both cell lines, suggest that the RIβ-L50R protein is protected from aggregation when the heterodimer RIβ-L50R:Cα complex forms.

### cAMP induces RIβ-L50R protein aggregation

**Figure 4:**
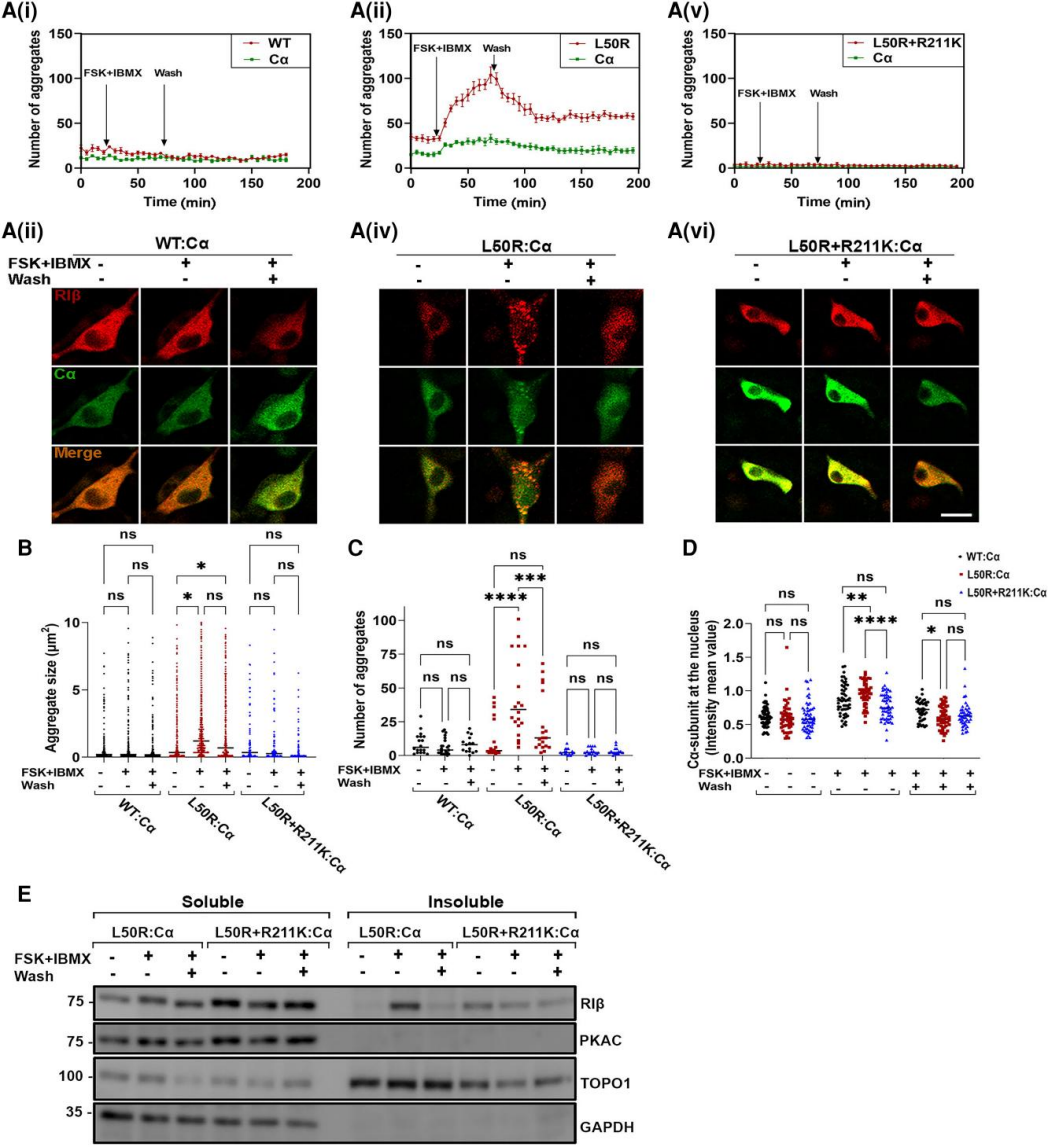
**Protein aggregation is dependent on cAMP concentration in the cell**. [**A**(**i**–**vi**)] Live cell imaging of rat pheochromocytoma (PC12) cells co-transfected to express catalytic subunit-α (Cα) and RIβ-wild-type (RIβ-WT) (**i** and **ii**), Cα and RIβ-L50R (**iii** and **iv**) or Cα and RIβ-L50R+R211K (**v** and **vi**). Cells were treated with 20 mM forskolin and 200 mM IBMX. Cells were imaged for 30 min before treatment, 30 min after treatment, and 60 min after washing the treatment. The number of aggregates was counted in RIβ-WT or mutants and Cα-expressing cells (**i**, **iii** and **vi**) over the course of the whole experiment. Images at three indicated time points taken from the live experiment are shown in **ii**, **iv** and **vi**, respectively. Scale bar = 20 mm. (**B**) Aggregate sizes (mm^2^) in cells expressing RIβ-WT or mutants were measured in ∼70 cells per condition. One-way ANOVA with Tukey’s *post hoc* honest significant difference (HSD) test. *P < 0.05; ns = non-significant comparison. (**C**) The number of aggregates were counted for each condition. One-way ANOVA with Tukey’s *post hoc* HSD test. **P < 0.01, ****P < 0.0001. (**D**) Nuclear mean fluorescence intensity to (cytoplasmic mean fluorescence intensity + nuclear mean fluorescence intensity) ratio was calculated for C-subunit. One-way ANOVA with Tukey’s *post hoc* HSD test. (**E**) Cell lysates of co-transfected cells expressing the indicated mutants were divided into soluble and insoluble fractions. GAPDH and TOPO1 were used as loading controls for the soluble and insoluble fractions, respectively. Cells transfected with plasmids with the indicated mutations were expressed similar levels of proteins for each treatment.

To assess whether cAMP induces protein aggregation upon RIβ-L50R:C complex dissociation, we performed live cell imaging. Accordingly, PC12 cells were co-transfected to express mCerulean-Cα and MKO2-WT or mutant RIβ and aggregation formation was monitored by confocal microscopy by following RIβ:C dissociation and reassociation (Fig. 4). Cells were treated with FSK, an adenylyl cyclase activator, and IBMX, a non-specific phospho-diesterase inhibitor able to prevent cAMP breakdown. The number of aggregates was counted before treatment (baseline), after treatment with FSK + IBMX, and following a long wash to allow restoration of cAMP levels to normal and complex reassociation. In cells co-expressing RIβ-WT:Cα, the FSK + IBMX treatment induced no detectable changes in the numbers or sizes of aggregates over the course of the experiment [Fig. 4A(i and ii), B and C]. However, significant increases in the number and size of RIβ aggregates were observed in cells co-expressing RIβ-L50R:Cα following FSK + IBMX treatment [Fig. 4A(iii and iv), B and C].

Interestingly, elevated cAMP levels induced RIβ-L50R aggregation, whereas Cα was still able to translocate into the nucleus [Fig. 4A(iv) and D]. A significant increase in the intensity mean value of Cα-subunit at the nucleus was calculated for RIβ-L50R:Cα-forming cells compared to RIβ-WT:Cα-forming cells following FSK + IBMX treatment (Fig. 4D). Such nuclear translocation was observed at 30 min after treatment of the RIβ-L50R:Cα-forming cells and 1 h after treatment of cells assembling the tetrameric RIβ-WT:Cα complex [Fig. 4A(ii and iv)].

Thirty minutes following FSK + IBMX treatment, the cells were washed for 1 h to reduce cAMP levels and allow the analysis of aggregation reversibility. The number of RIβ-L50R aggregates was significantly reduced after washing the FSK + IBMX treatment and reducing cAMP levels (Fig. 4C). However, the sizes of aggregates were significantly larger than seen before treatment (Fig. 4B). Thus, the results suggest that reducing cAMP levels reverses protein aggregation by allowing heterodimer formation.

To examine cAMP-induced protein aggregation, we introduced a mutation resulting in R211K replacement into the RIβ-L50R construct. The R211 is located in the cAMP binding site and thus a replacement to a Lys reduces the affinity of the regulatory subunit to cAMP. Therefore, we expected that the RIβ-L50R + R211K:Cα complex would have a reduced sensitivity to cAMP. Before conducting the live imaging experiment, we verified that introducing the R211K mutation into the RIβ-L50R mutant protein still results in a protein that cannot homodimerize (Supplementary Fig. 8). A live imaging experiment showed that the RIβ-L50R + R211K:Cα complex indeed did not respond to elevated cAMP levels and that Cα did not translocate into the nucleus [Fig. 4A(v and vi)]. The RIβ-L50R + R211K protein showed no significant change in aggregation formation (i.e. numbers or sizes of aggregates) during the live imaging experiment [Fig. 4A(v and vi), B and C]. This further demonstrates the protective role of the RIβ:C heterodimer against protein aggregation.

Finally, to analyse protein aggregates following cell treatments biochemically, we performed a western blot analysis on the cell lysates of transfected cells expressing either RIβ-L50R:Cα or RIβ-L50R + R211K:Cα complexes. Cell lysates were separated for soluble and insoluble fractions prior to FSK + IBMX treatment, after 30 min of treatment and following a 60 min wash. Consistent with the aggregation observed in the live cell images, RIβ-L50R was detected in the insoluble fraction after FSK + IBMX treatment and found in the soluble fraction after the washes. This suggests that protein aggregation is reversible and depends on cAMP levels. RIβ-L50R + R211K remained in soluble fraction with no changes observed following FSK + IBMX treatment, as observed with RIβ-L50R, suggesting that the R211K replacement protects RIβ from aggregation and C-subunit nuclear translocation by maintaining the interactions of the heterodimer (Fig. 4D and E).

### RIβ-L50R aggregation is dependent on the RIβ:C ratio in the cell

**Figure 5:**
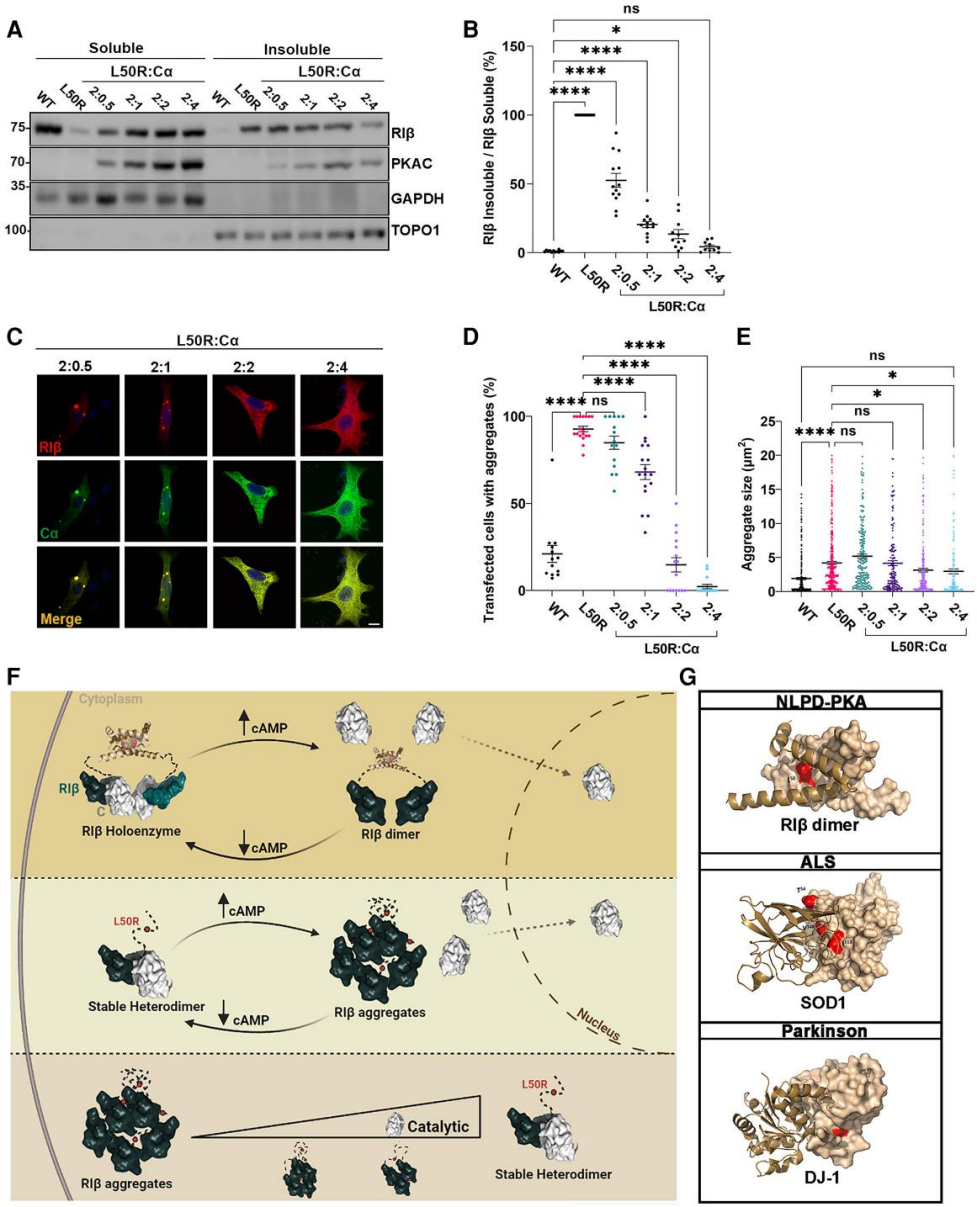
**RIβ aggregation formation is inhibited by increasing C-subunit protein expression levels**. (**A**) PC12 cells were transfected to express RIβ-wild-type (WT) or RIβ-L50R alone to compare protein solubility. The RIβ-L50R-encoding plasmid was co-transfected with increasing amounts of the Cα-subunit-encoding plasmid at the indicated ratios. Protein lysates were divided to soluble and insoluble fractions, separated by sodium dodecyl-sulfate polyacrylamide gel electrophoresis (SDS-PAGE) and immunoblotted with specific antibodies. GAPDH and TOPO1 were used as loading controls for the soluble and insoluble fractions, respectively. (**B**) Quantitative analysis of western blot intensities. Lysates from three independent experiments underwent multiple SDS-PAGE runs. Each dot represents the ratio of RIβ-L50R insoluble divided by RIβ-L50R soluble from distinct gel runs. One-way ANOVA with Dunnett’s *post hoc* test. (**C**) Representative confocal images of PC12 cells co-transfected to generate the indicated RIβ:C ratios. RIβ (red); Cα (green). Scale bar = 20 mm. (**D**) Quantification of transfected cells with aggregates at indicated RIβ-L50R:Ca ratios from total cells (%) from 132 cells for RIβ WT, 154 for L50R, 119 for a 2:0.5 ratio, 112 for a 2:1 ratio, 117 for a 2:2 ratio and 142 for a 2:4 ratio. Each dot in the graph represent % transfected cells with aggregates in an image taken at ×20. Statistics from three independent experiments. One-way ANOVA with Dunnett’s *post hoc* test. ****P < 0.00001; ns = non-significant comparison. (**E**) Same images as in **D** quantified for aggregate size (mm^2^) at the indicated RIβ-L50R:Cα ratios. One-way ANOVA with Tukey’s *post hoc* honest significant difference test. (**F**) Schematic illustration that emphasizes the differences between the healthy and disease state regarding RIβ dimerization, catalytic subunit binding, protein aggregation, and nuclear translocation in response to cAMP levels. (**G**) Dimers of the RIβ dimerization and docking (D/D) domain, SOD1 and DJ-1 proteins (Protein Data Bank accession numbers are: 4F9K, 1HL4 and 1J42, respectively). One protomer is depicted as a surfaces representation, while the second protomer is represented by helix structures. Red surfaces indicate the location of identified mutations at the dimer interface. Residues T54, I113 and V148 are shown in SOD1. L166 is shown in DJ-1.

The fact that we observed RIβ aggregates in human patient samples (Fig. 1), together with the findings that the Cα-subunit protects RIβ-L50R from self-aggregation when co-expressed in tissue culture cells (Figs 3 and 4), led us to hypothesize that RIβ-L50R protein aggregation depends on Cα-subunit protein expression levels. To test this hypothesis, we transiently transfected PC12 cells to express RIβ-L50R in the absence of the Cα-subunit or the presence of increasing concentrations of the Cα-subunit. Cell lysates were separated into soluble and insoluble fractions, which were resolved on SDS-PAGE to determine RIβ solubility. As expected, RIβ WT proteins were found in the soluble fraction, while RIβ-L50R proteins accumulated in the insoluble fraction when expressed without the Cα-subunit. Increasing the Cα-subunit expression levels gradually increased the proportion of RIβ subunits in the soluble fraction relative to its presence in the insoluble fraction, thus promoting RIβ protein solubility (Fig. 5A). The graph in Fig. 5B demonstrates the gradual decrease in the ratio of insoluble RIβ:soluble RIβ that is correlated with the increase in Cα-subunit protein expression levels, as determined by assessing the intensity of the relevant western blot bands.

Consistent with these biochemical assays, we observed fewer RIβ aggregates in cells co-transfected to express RIβ when Cα-subunit expression was increased. An RIβ:C ratio of 2:2 (or 1:1) was sufficient to reduce protein aggregation to the level seen in the WT protein (Fig. 5C and E). These results demonstrated how a gradual increase in Cα-subunit expression protected RIβ mutant protein from self-aggregation. To verify that the protective effect of Cα-subunit expression on protein aggregation was specific and did not result from a sequestering of RIβ-self aggregation due to the expression of another protein, we over-expressed RIβ with the mitochondrial AKAP1 (dAKAP1). Co-expression of RIβ WT and dAKAP1 resulted in the recruitment of RIβ to the mitochondria (Supplementary Fig. 9A). Increasing dAKAP1 expression levels together with co-expression with RIβ-L50R decreased the sizes of aggregates but did not change the number of transfected cells with aggregates, compared to the number of aggregates seen with RIβ L50R expression alone (Supplementary Fig. 9B and C).

In summary, in the healthy state, the PKA RIβ-specific holoenzyme consists of a dimer of two heterodimers, each composed of RIβ and C-subunits. RIβ:RIβ homodimers bind to two C-subunits to form two (RIβ:C) heterodimers. When cAMP levels increase in the cell, RIβ:RIβ homodimers dissociate from the C subunits, allowing the C-subunits to translocate into the nucleus (Fig. 5F, top). In the disease state, the RI-L50R mutant protein is unable to form RIβ:RIβ homodimers but can still bind to the C subunit, forming RIβ:C heterodimers. When cAMP levels are elevated, heterodimer dissociation occurs, leading to RIβ-L50R protein aggregation. However, the C subunit is still capable of translocating into the nucleus (Fig. 5F, middle). As illustrated, the formation of RIβ:C heterodimers protects the RIβ-L50R protein from aggregation. Protein aggregation is influenced by cAMP concentrations in the cell and the ratio of RIβ:C proteins (Fig. 5F, bottom).

Structural analysis of proteins harbouring familial mutations causing protein misfolding in neurodegenerative disease has revealed a common mechanism involving dimer destabilization and monomer aggregation (Fig. 5G). Mutations affecting, for example, the superoxide dismutase 1 gene (*SOD1*) in amyotrophic lateral sclerosis (ALS) or DJ-1 [also known as parkinsonism associated deglycase (PARK7)] in Parkinson’s disease are located at the dimer interface, leading to dimer destabilization and subsequent protein aggregation. These findings underscore the significance of understanding the shared mechanisms underlying protein misfolding and aggregation in diverse neurodegenerative diseases.

## Discussion

Our findings revealed the pathophysiological mechanism for a rare neurodegenerative disease that is driven by a missense mutation in the PKA RIβ regulatory subunit. A mouse model expressing the RIβ-L50R mutant protein exhibits altered motor performance, allowing us to define this mutation as an evolutionarily conserved driver mutation for protein aggregation and neuronal loss across species. While the behavioural phenotype in mice manifests at an older age, the molecular, biochemical and cellular changes driven by the mutation start at a younger age. This finding suggests that the underlying pathological process occurs earlier, even before the onset of observable symptoms. Moreover, this study provides clinical evidence for RIβ regulatory subunit dimerization, a molecular signature of all PKA regulatory subunits, which has been studied *in vitro* for decades, as well as a clinically relevant mutation that interferes with dimerization formation, leading to monomer aggregation and neurodegeneration. The C-subunit serves as a safeguard for RIβ-L50R protein stability by interacting and forming the RIβ-L50R:C heterodimer. Furthermore, introducing a mutation that reduces RIβ affinity to cAMP and thus reduces heterodimer dissociation, protecting against cAMP induced RIβ-L50R aggregation. The formation of a heterodimer instead of ‘a dimer of heterodimers’ not only prevents PKA anchoring by AKAPs but also leads to altered allosteric regulation.

PKA activity plays a neuroprotective role that is critical for many neuronal functions.^42^ Dimerization of the regulatory subunits is a prerequisite for kinase anchoring near dedicated substrates.^13,16^ We previously demonstrated how each PKA regulatory subunit is assembled into a distinct quaternary structure when interacting with the same C-subunit.^8^ These isoform-specific PKA holoenzyme structures differ in terms of their allosteric regulation and interactions with AKAPs. While we and others have extensively studied the rationale for isoform non-redundancy and the tightly controlled allosteric regulation in physiological conditions, no pathophysiological changes, observed in patients, were associated with PKA holoenzyme dis-assembly. The RIβ-L50R mutation not only interferes with homodimerization but also eliminates the formation of a dimer of heterodimers. Consequently, the PKA holoenzyme exists as one RIβ-L50R:C heterodimer and cannot bind AKAPs since the docking site created by formation of the homodimerization is absent. We further showed that the RIβ-L50R heterodimer formation is reversible, with dissociation and reassociation depending on cAMP concentration in the cell. Following reassociation, the number of aggregates is significantly decreased, suggesting aggregation reversibility, although the sizes of the remaining aggregates become larger. Recently, Zhang *et al.*^43^ demonstrated that the RIa subunit can form phase-separated condensates, termed dynamic RIa puncta, which increase in number upon cAMP elevation, resembling the RIβ aggregates observed in our study. We designate these RIβ aggregates in this article for several reasons. Firstly, these RIβ mutant proteins are found in insoluble fractions, indicating a redisposition to aggregation. Additionally, analysis using aggrescan software identified aggregation-prone segments within the D/D domain sequence, which, when replaced with the L50R mutation, exhibit an increased tendency to aggregate due to disruption of protomer dimerization and exposure of hydrophobic residues. Moreover, the term ‘aggregates’ typically refers to the formation of larger and pathological assemblies of molecules. Finally, the presence of RIβ aggregates in various brain regions of patients suffering from a neurodegenerative disease underscores their characterization as aggregates, as protein aggregation is the hallmark signature of such conditions.

We also considered whether the RIβ-L50R:C heterodimer would be less allosterically regulated than the RIβ_2_:C_2_ tetramer and would thus dissociate faster upon increased cAMP concentration or whether the mutation creates a structural conformation that prevents or slows the heterodimer dissociation. We found that the C-subunit intensity at the nucleus following RIβ-L50R:C complex dissociation is significantly higher than the C-subunit intensity at the nucleus following RIβ_2_:C_2_ complex dissociation, suggesting a reduced PKA holoenzyme allostery and potential implications for cellular processes. Given the critical role of PKA in phosphorylating transcription factors and chromatin-modifying enzymes, the elevated presence of C-subunits in the nucleus may exert pronounced effects on gene expression profiles.

To validate that aggregation formation is a result of cAMP binding and heterodimer dissociation, we introduced the R211K modification into the RIβ-L50R construct to reduce cAMP binding affinity. We showed that the RIβ-L50R protein that is defective in cAMP binding did not aggregate upon FSK/IBMX treatment.

The RIβ-L50R:C heterodimer assumes a stable conformation that probably sequesters the sequence in the R-subunit dimerization domain that is prone to aggregation, such that the C-subunit plays a protective role against aggregation. PKA R-subunits are multi-domain proteins that include the D/D domain, which forms a stable and independent conformation, followed by a flexible linker that contains the inhibitor site and two cAMP-binding domains in tandem. We showed that the flexible linkers are major factors in promoting different inter-domain arrangements that lead to distinct isoform-specific quaternary structures. Protein dimerization can be realized by several mechanisms. In all R-subunits, the D/D domain is an isologous dimer, i.e. the same binding sets of the two subunits (protomers) complete one another and are rotated 180° relative to one another. The stability of this dimeric structure relies on specific amino acid residues at the interface between the two subunits, which prevent exposure of hydrophobic residues. However, in the disease state, a Leu to Arg substitution disrupts these stabilizing interactions. Our *in silico* analysis predicted that this substitution causes steric clashes with the surrounding residues, and the positively large Arg residue cannot fit within this region. Consequently, the dimerization fails to fold properly, resulting in exposure of hydrophobic residues and promoting aggregation of the RIβ-L50R proteins.

The rare neurodegenerative disease addressed here is a member of an increasingly common class of neurological disorders characterized by aggregation of proteins assuming aberrant conformations. By deciphering the molecular mechanism leading to PKA RIβ aggregation, we shed light on a common mechanism of dimer destabilization across neurodegenerative diseases. In various neurodegenerative conditions, there are several familial mutations that affect the dimer interface of various affected proteins, which are associated with monomer aggregation. Indeed, more than 90 mutations in SOD1 are linked to ALS, a neurodegenerative disorder predominantly affecting upper and lower motor neurons.^44,45^ SOD1 protein is expressed as a homodimer in the cytosol. Many of the familial mutations in SOD1, affecting the dimer interface, impair dimerization and, subsequently, lead to protein aggregation.^46,47^ Destabilization of the dimer interface has also been suggested in the PD-associated L166P, M26I and L10P mutants of DJ-1, with the loss-of-function or inactivation of DJ-1 having been assigned to monomerization after such destabilization.^48-52^ In general, interface regions are more prone to aggregate than other surface regions. The interactions that promote the formation of dimerization are hydrophobic. Therefore, exposure of these hydrophobic residues leads to abnormal intermolecular aggregation and protein misfolding. Specific interactions between proteins that are prone to aggregation and a binding partner can attenuate protein aggregation. For example, DJ-1 interactions with α-synuclein attenuate aggregation and cellular toxicity in models of Parkinson’s disease.^53^ Macrophage migration inhibitory factor (MIF) similarly reduces the amount of misfolded mutant SOD1 through a direct interaction.^54^ In the PKA neurodegenerative disease considered here, based on PKA-associated mutation, binding of the C-subunit prevents RIβ-L50R aggregation in a dose-dependent manner. Accordingly, we showed that the expression levels of RIβ proteins were higher in patients that carry the L50R mutation, when compared to healthy individuals, while the expression levels of the C-subunit were similar in both groups and across different brain regions. This suggests that the molecular balance between levels of the regulatory and C-subunits is critical for RIβ protein misfolding.

It is also notable that our human and mouse findings overlap in the cerebellum. We found atrophy of folium IX in the cerebellum of the L50R mouse model, and this reflects the cerebellar atrophy in the individuals with the L50R mutation. In recent years, studies have caused us to reconsider the role of the cerebellum and appreciate that it is not just involved in motor control but also cognition and affective functions.^55^ Specifically, underactivation of left lobule IX may be implicated in attention and hand-eye coordination,^56^ behaviours that are dysregulated in neurodegeneration. Furthermore, cortical atrophy of lobule IX has been associated with attention deficit hyperactivity disorder^57^ and frontotemporal dementia.^58^

Recent research on the stoichiometry of PKA has revealed that RI and RII subunits greatly outnumber the C subunits across various rat tissues, with the possibility of existing without any C subunit bound even at the basal level of cAMP. Particularly striking is the cerebellum where RI subunits are exceptionally abundant, surpassing C subunits by approximately 17-fold.^59^ In Fig. 5, we demonstrate the critical importance of the ratio between RIβ and Cα subunits in preventing RIβ aggregation, showing that an excess of RIβ mutant leads to protein aggregation. Consequently, in the cerebellum, the skewed molecular ratio suggests that C subunit binding might have a limited effect on RIβ aggregation. This finding could explain why a point mutation in RIβ specifically affects cerebellum and motor function.

In summary, we have identified the molecular mechanism leading to a novel neurodegenerative disease driven by a mutation in the PKA regulatory subunit RIβ. This study sheds light on dimerization interference as a molecular mechanism for protein misfolding that occurs in several neurodegenerative diseases. Understanding the structural changes caused by the mutation and subsequent interaction with a stabilizing protein can provide insights into the development of therapies aimed at stabilizing the conformation of a mutant protein.

## Supplementary Material

awae154_Supplementary_Data

## Data Availability

Biochemical and cellular imaging data were acquired from both animal models and human samples at the Ilouz Lab, Bar Ilan University, Israel. The animal model was generated at University of Iowa, with behavioural studies conducted there, as demonstrated in Fig. 1O. The PCA assay shown in Fig. 3A was performed at the University of Innsbruck. Derived data supporting the findings of this study are available from the corresponding author upon request.
