# Supplementary material for: A mutation in the PRKAR1B gene drives pathological mechanisms of neurodegeneration across species
Source: Brain. 2024 May 14;147(11):3890–905. doi: 10.1093/brain/awae154 (PMC11531844; doi:10.1093/brain/awae154)
Supplement: awae154_Supplementary_Data [file awae154_supplementary_data.zip › brain-2023-02204-File007.pdf]

**Supplementary Figure 1 (for main Fig. 1) Brain atrophy quantifications from a MRI of a newly diagnosed patient that carry the RIβ-L50R mutation. A.** Parietal right lobe. **B.** Parietal left lobe. **C.** Frontal right lobe. **D.** Frontal left lobe. **E.** Occipital left lobe. Curves defined by %Intracranial Volume (%ICV) scores plotted against the reference centile curves in normal population. Red dot represents the ICV of the newly diagnosed patient relative to the normal volume.

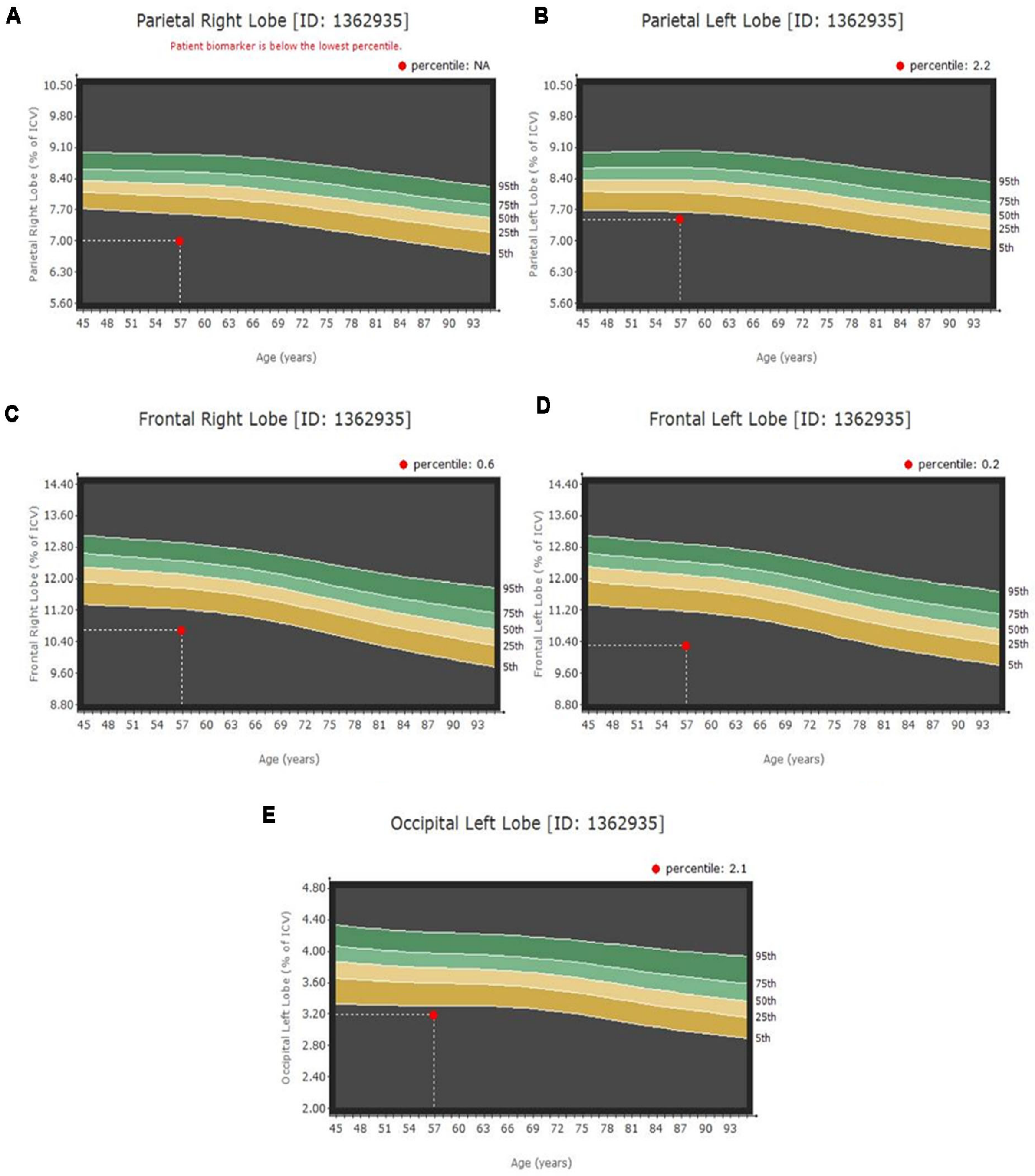
A
Parietal Right Lobe [ID: 1362935]
Patient biomarker is below the lowest percentile.
percentile: NA
Parietal Right Lobe (% of ICV)
10.50
9.80
9.10
8.40
7.70
7.00
6.30
5.60
95th
75th
50th
25th
5th
45 48 51 54 57 60 63 66 69 72 75 78 81 84 87 90 93
Age (years)
B
Parietal Left Lobe [ID: 1362935]
percentile: 2.2
Parietal Left Lobe (% of ICV)
10.50
9.80
9.10
8.40
7.70
7.00
6.30
5.60
95th
75th
50th
25th
5th
45 48 51 54 57 60 63 66 69 72 75 78 81 84 87 90 93
Age (years)
C
Frontal Right Lobe [ID: 1362935]
percentile: 0.6
Frontal Right Lobe (% of ICV)
14.40
13.60
12.80
12.00
11.20
10.40
9.60
8.80
95th
75th
50th
25th
5th
45 48 51 54 57 60 63 66 69 72 75 78 81 84 87 90 93
Age (years)
D
Frontal Left Lobe [ID: 1362935]
percentile: 0.2
Frontal Left Lobe (% of ICV)
14.40
13.60
12.80
12.00
11.20
10.40
9.60
8.80
95th
75th
50th
25th
5th
45 48 51 54 57 60 63 66 69 72 75 78 81 84 87 90 93
Age (years)
E
Occipital Left Lobe [ID: 1362935]
percentile: 2.1
Occipital Left Lobe (% of ICV)
4.80
4.40
4.00
3.60
3.20
2.80
2.40
2.00
95th
75th
50th
25th
5th
45 48 51 54 57 60 63 66 69 72 75 78 81 84 87 90 93
Age (years)

**Supplementary Figure 2 (for main Fig. 1). A.** Generation off RIβ-L50R heterozygous mouse model. **A(i).** The L50R missense mutation was introduced into the PrkarIb gene by CRISPR/Cas9 technology, generating three independent strains. **A(ii).** Representative image of sanger sequencing was used to identify the presence of the mutation. **A(iii).** Schematic of the specific nucleotide substitution and corresponding amino acid change in the mouse model. **B.** The L50R mutation in the mouse model did not affect gene expression of PKA subunits as compared to the expression levels in WT littermates. Gene expression of PrkarIb (RIβ), PrkarIa (RIα), PrkarIIa (RIIα), PrkarIIb (RIIβ), Prkarca (Cα), and Prkarcb was quantified in Hippocampus **(B(i))**, Cerebellum **(B(ii))**, and Striatum **(B(iii))** samples from old male mice. Expression levels of Dnaaf1 and Pdgfa were also analyzed due to their close proximity to the L50R mutation. Their expression levels did not change in the presence of the L50R mutation.

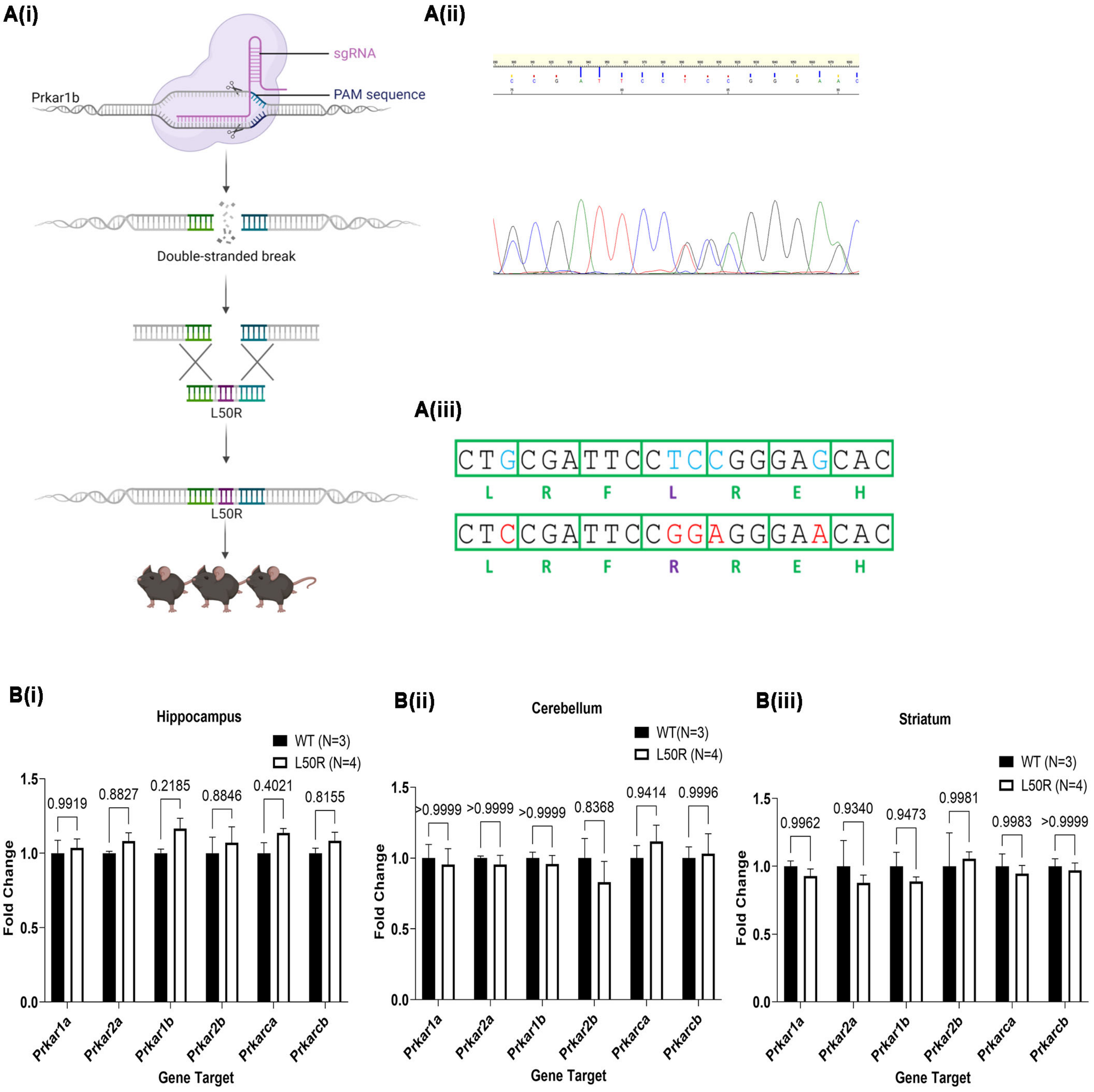
A(i)
sgRNA
PAM sequence
Prkar1b
Double-stranded break
L50R
L50R
A(ii)
A(iii)
CTG CGA TTC CTC CGG GAG CAC
L R F L R E H
CTC CGA TTC CGG AGG GAA CAC
L R F R R E H
B(i)
Hippocampus
WT (N=3)
L50R (N=4)
0.9919
0.8827
0.2185
0.8846
0.4021
0.8155
Fold Change
Gene Target
Prkar1a
Prkar2a
Prkar1b
Prkar2b
Prkarca
Prkarcb
B(ii)
Cerebellum
WT(N=3)
L50R (N=4)
>0.9999
>0.9999
>0.9999
0.8368
0.9414
0.9996
Fold Change
Gene Target
B(iii)
Striatum
WT (N=3)
L50R (N=4)
0.9962
0.9340
0.9473
0.9981
0.9983
>0.9999
Fold Change
Gene Target

**Supplementary Figure 3 (for main Fig. 1) Behavioral testing of the L50R mouse model identified age-dependent motor performance changes and hyperactivity only. A.** The L50R mutation does not cause motor performance changes in young mice (WT *n*=3; L50R *n*=4) as determined by the accelerating rotarod test. Repeated measures ANOVA with Šídák correction for multiple testing. **B.** Activity monitoring testing found hyperactivity in L50R old mice (WT *n*=4; L50R *n*=5) with a time X genotype *P* = 0.0099. Repeated measures ANOVA with Šídák correction for multiple testing. **C.** The L50R mutation does not impact spatial learning and memory in old mice (WT *n*=13; L50R *n*=8) as determined by the barnes maze test. Repeated measures ANOVA with Šídák correction for multiple testing. **D.** The L50R mutation does not impact learning and memory in aged mice (WT *n*=10; L50R *n*=18) as determined by fear conditioning (0.75mA). Repeated measures ANOVA with Šídák correction for multiple testing. **E.** The elevated zero maze test found no anxiety-like behaviors associated with the L50R mutation in old mice (WT *n*=17; L50R *n*=17). Unpaired t test. **F.** The L50R mutation does not impact social interaction in old mice (WT *n*=10; L50R *n*=19) as determined by the three-chamber social preference test. Repeated measures ANOVA with Šídák correction for multiple testing. In all graphs each dot represents a mouse except the graph in B where each dot is the average number of *n*.

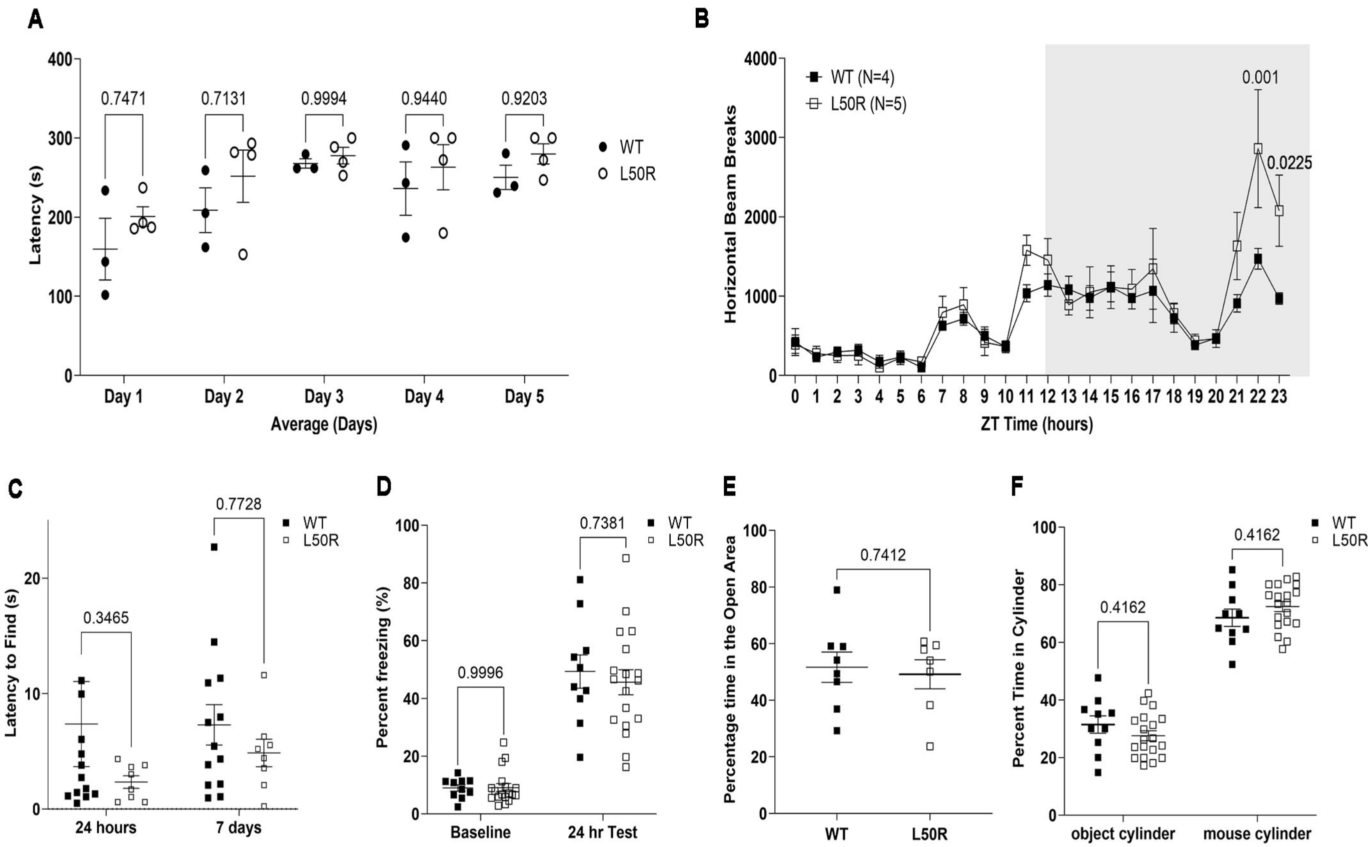
A
Latency (s)
400
300
200
100
0
0.7471
0.7131
0.9994
0.9440
0.9203
Day 1
Day 2
Day 3
Day 4
Day 5
Average (Days)
WT
L50R
B
Horizontal Beam Breaks
4000
3000
2000
1000
0
WT (N=4)
L50R (N=5)
0.001
0.0225
0 1 2 3 4 5 6 7 8 9 10 11 12 13 14 15 16 17 18 19 20 21 22 23
ZT Time (hours)
C
Latency to Find (s)
20
10
0
0.3465
0.7728
24 hours
7 days
WT
L50R
D
Percent freezing (%)
100
80
60
40
20
0
0.9996
0.7381
Baseline
24 hr Test
WT
L50R
E
Percentage time in the Open Area
100
80
60
40
20
0
0.7412
WT
L50R
F
Percent Time in Cylinder
100
80
60
40
20
0
0.4162
0.4162
object cylinder
mouse cylinder
WT
L50R

**Supplementary Figure 4 (for main Fig. 1) The RIβ is prone to aggregation in the PSAPP Alzheimer's disease mouse model. A-B.** Slices from hippocampi focusing on CA1 regions from WT (upper panel) or PSAPP young mice (4 months old) **(A)** and old mice (13 months old) **(B)** immuno-stained with anti-RIβ (red) or anti-PKACβ (green) antibodies. Protein aggregations are denoted by white arrows. The images were acquired by a Zeiss LSM 780 confocal microscope with X20 lenses. Scale Bar: 50 µm; Zoom-in image: 25 µm. **C-F.** Paired comparison analyses of number of aggregates per area unit was calculated and compared as denoted in each graph. Each dot presents an aerial unit. Error bars represent ±SEM, unpaired t test. ****$P<0.0001$, ns; non-significant comparison.

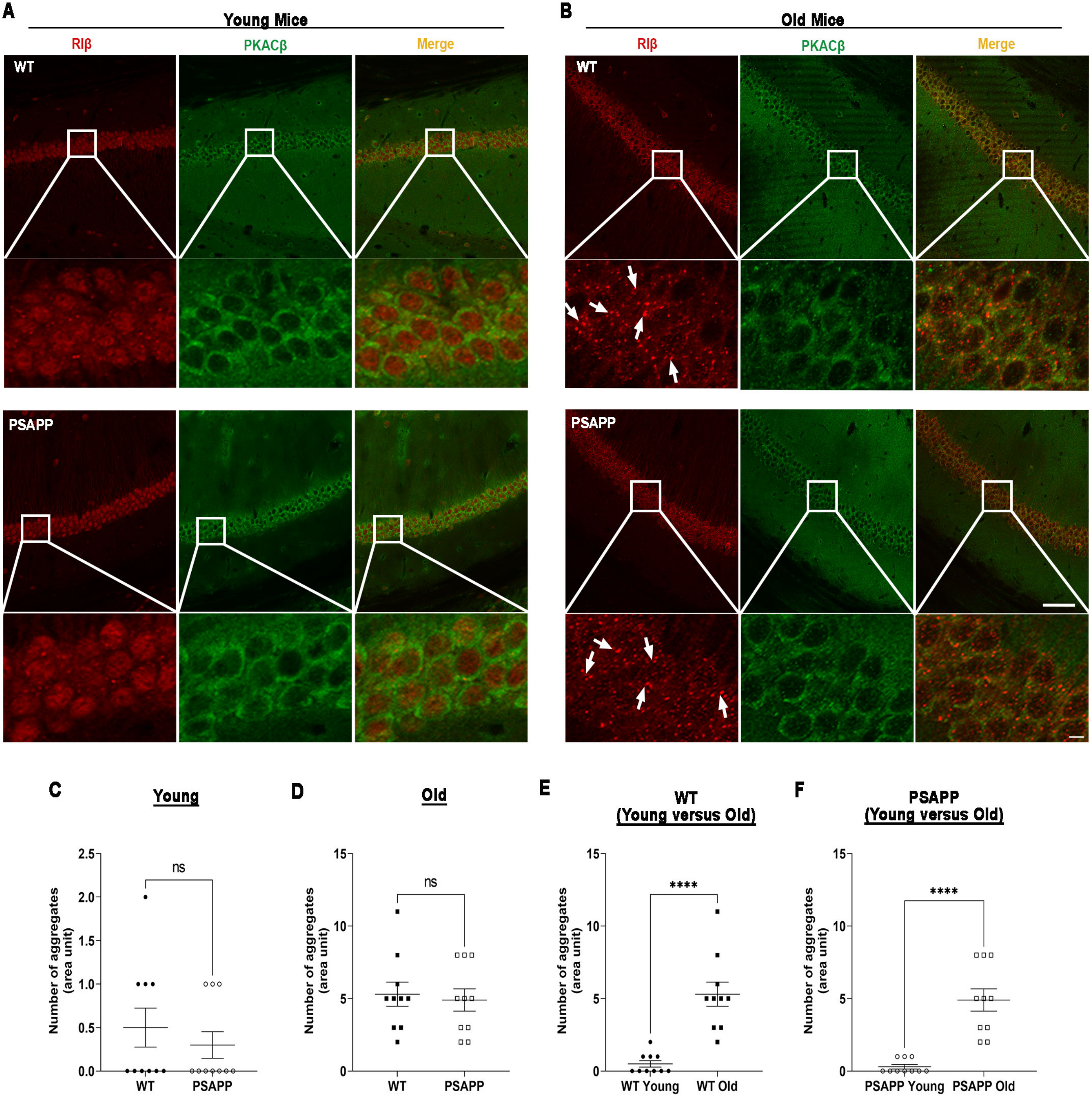
A
Young Mice
RIβ
PKACβ
Merge
WT
PSAPP
B
Old Mice
RIβ
PKACβ
Merge
WT
PSAPP
C
Young
ns
Number of aggregates (area unit)
2.5
2.0
1.5
1.0
0.5
0.0
WT
PSAPP
D
Old
ns
Number of aggregates (area unit)
15
10
5
0
WT
PSAPP
E
WT (Young versus Old)
****
Number of aggregates (area unit)
15
10
5
0
WT Young
WT Old
F
PSAPP (Young versus Old)
****
Number of aggregates (area unit)
15
10
5
0
PSAPP Young
PSAPP Old

**Supplementary Figure 5 (for main Figure 2) Protein aggregates in human samples contains RIβ-L50R proteins but not the PKA C-subunit, which remains diffuse in the cytoplasm. A.** RIβ domain organization. D/D domain, Inhibitor sequence (IS), cAMP binding domains (CNB-A, CNB-B). Multiple sequence alignment of the RIβ D/D domain from different organisms. The L50 that is conserved across all sequences is highlighted in purple. The conserved Cys residues that form inter-disulfide bonds between the protomers are highlighted in yellow. Predicted aggregation-prone segments in the D/D domain sequence are in red boxes (http://bioinf.uab.es/aggrescan/). **B-E.** Paraffin-embedded human tissues from the amygdala **(B)** substantia nigra **(C)** thalamus **(D)** and cerebellum **(E)** Immuno-stained with anti-RIβ or PKA-Cβ antibodies. PKA RIβ-L50 (healthy individual), RIβ -L50R (patient with the heterozygous mutation), PKAC subunit. In the amygdala, 84% of cells presented with aggregates (496/590 cells). In the substantia nigra, 81% of the cells counted with aggregates (579/710 cells). In the thalamus, 83% of the cells counted with aggregates (453/540 cells). In the cerebellum, 51% of the cell counted with aggregates (435/845). Scale bar: 20 µm; Zoom-in image: 20 µm.

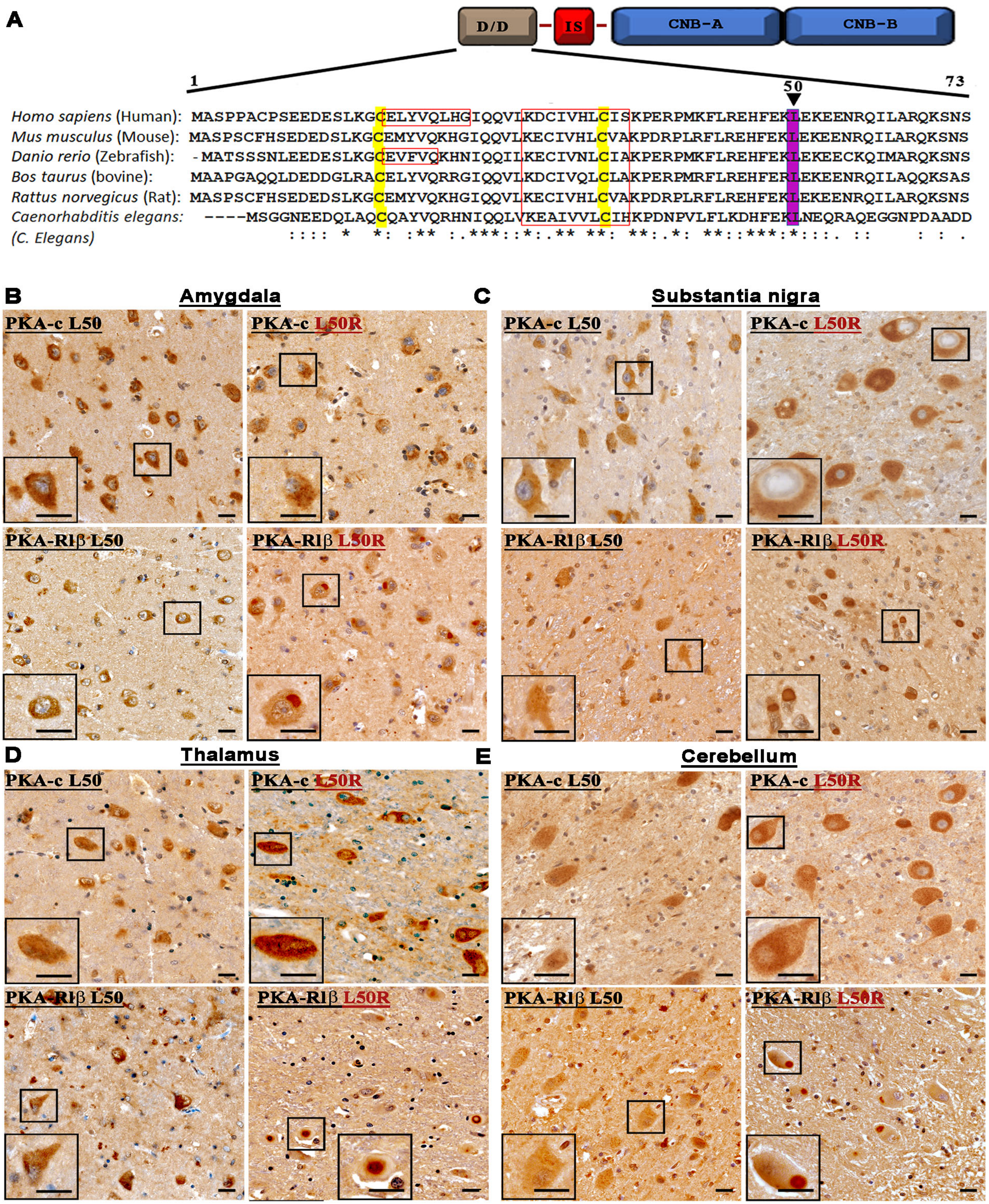

A
D/D
IS
CNB-A
CNB-B
1
50
73
Homo sapiens (Human): MASPPACPSEEDESLKGCELYVQLHGIQQVLKDCIVHLCISKPERPMKFLREHFEKLEKEENRQILARQKSNS
Mus musculus (Mouse): MASPSCFHSEDEDSLKGCEMYVQKHGIQQVLKECIVHLCVAKPDRPLRFLREHFEKLEKEENRQILARQKSNS
Danio rerio (Zebrafish): -MATSSSNLEEDESLKGCEVFVQKHNIQQILKECIVNLCIAKPERPMKFLREHFEKLEKEECKQIMARQKSNS
Bos taurus (bovine): MAAPGAQQLDEDDGLRACELYVQRRGIQQVLKDCIVQLCLAKPERPMRFLREHFERLEKEENRQILAQQKSAS
Rattus norvegicus (Rat): MASPSCFHSEDEDSLKGCEMYVQKHGIQQVLKECIVHLCVAKPDRPLRFLREHFEKLEKEENRQILARQKSNS
Caenorhabditis elegans: ----MSGGNEEDQLAQCQAYVQRHNIQQLVKEAIVVLCIHKPDNPVLFLKDHFEKLNEQRAQEGGNPDAADD
(C. Elegans)
B
Amygdala
C
Substantia nigra
PKA-c L50
PKA-c L50R
PKA-RIβ L50
PKA-RIβ L50R
D
Thalamus
E
Cerebellum

**Supplementary Figure 6 (for main Figure 2) The protein localization changes observed in the RIβ-L50R mice start at a young age and remain with the age. A.** Immunohistochemical staining of cerebellum sagittal sections from wildtype (WT) and RIβ-L50R mutant young mice (3-4 months old). The sections were labeled with anti-RIβ (red) and anti-PKACβ (green) antibodies. Images were obtained with confocal microscope at X20 magnification. Scale Bar: 50 µm; White boxes zoom-in, scale bar: 10 µm. **B.** Total cell lysates of cerebellum from WT and RIβ-L50R mutant old mice (16-20 months old) were extracted and separated to soluble and insoluble fractions. Proteins were loaded on SDS-PAGE and probed with indicated antibodies. GAPDH and α-internexin were used as loading controls for soluble and insoluble fractions, respectively. **C-D.** Quantifications of band intensity of RIβ **(C)** or PKAC **(D)**. Total proteins and soluble fractions were normalized to GAPDH and insoluble fractions were normalized to α-internexin. Each dot represents lysates from a different mouse. Error bars represent ±SEM, unpaired t test. ***$P$<0.001, ****$P$<0.0001, ns; non-significant. **E.** Immunohistochemical staining of sagittal sections from WT and RIβ-L50R mutant old mice (16-20 months old). Images obtained described in A.

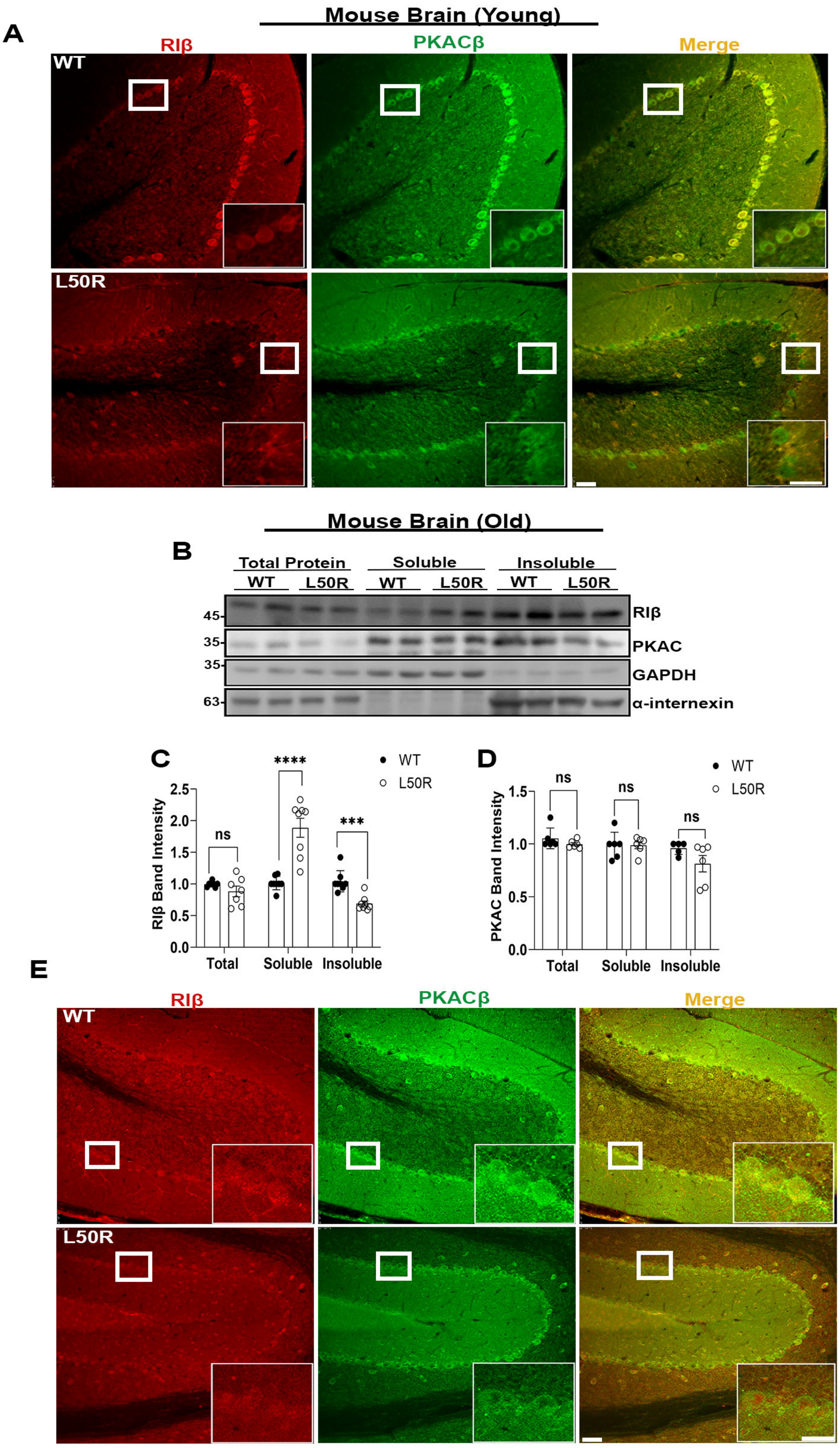
Mouse Brain (Young)
A
RIβ
PKACβ
Merge
WT
L50R
Mouse Brain (Old)
B
Total Protein
Soluble
Insoluble
WT
L50R
WT
L50R
WT
L50R
45-
35-
35-
63-
RIβ
PKAC
GAPDH
α-internexin
C
RIβ Band Intensity
2.5
2.0
1.5
1.0
0.5
0.0
ns
****
***
WT
L50R
Total
Soluble
Insoluble
D
PKAC Band Intensity
1.5
1.0
0.5
0.0
ns
ns
ns
WT
L50R
Total
Soluble
Insoluble
E
RIβ
PKACβ
Merge
WT
L50R

**Supplementary Figure 7 (for main Fig 3) A.** Confocal microscopy images of transiently transfected HEK293T cells using mKO2-tagged RIβ (WT or mutants). **B.** The percentage of transfected cells that contains aggregates. Approximately one hundred cells were counted for each transfection. **C-D.** Cell lysates of transiently transfected cells were divided into soluble **(C)** and insoluble **(D)** fractions and were separated by SDS-PAGE under reduced conditions (upper gel) or non-reduced conditions (lower gel). GAPDH was used as a loading control for soluble fraction. TOPO1 served as a loading control for the insoluble fraction. Scale bar: 20μm. **E.** HEK293T cells were co-transfected to express mKO2-tagged RIβ WT or RIβ mutants and mCerulean-Cα. Scale bar: 10 μm. **F.** Soluble lysate proteins of HEK293T cells were resolved by SDS-PAGE and immunoblotted with anti- RIβ or -Cα antibodies. GAPDH was used as a housekeeping protein loading control for each transfection. **G.** The same protein samples as in F separated by SDS-PAGE under non-reducing conditions and immunoblotted with RIβ antibody. RIβ monomers and dimers are noted. RIβ L50R, L50A and L50K proteins cannot dimerize, even when co-expressed with the Cα subunit.

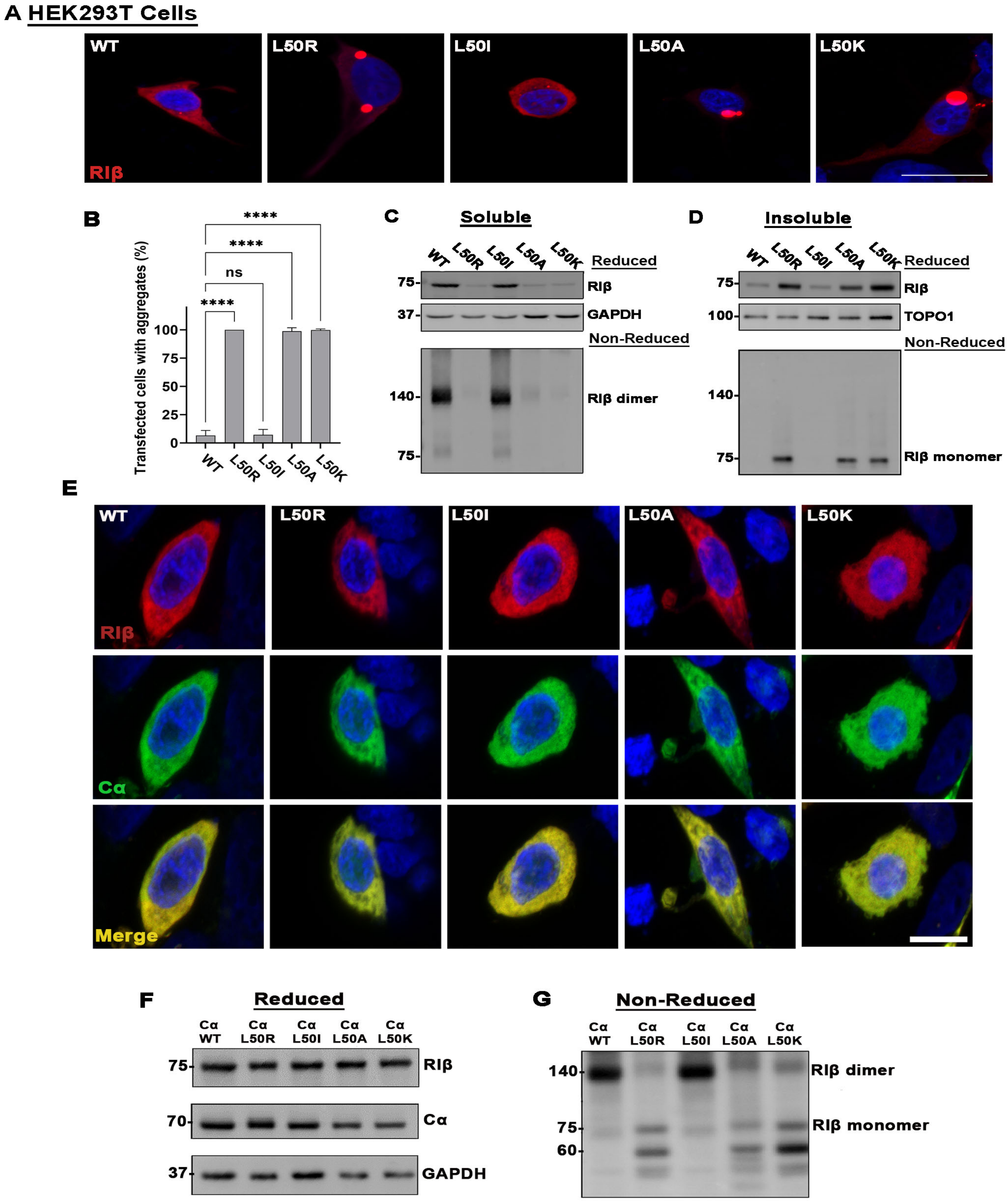
A HEK293T Cells
WT
L50R
L50I
L50A
L50K
RIβ
B
Transfected cells with aggregates (%)
100
75
50
25
0
****
ns
****
****
WT
L50R
L50I
L50A
L50K
C
Soluble
Reduced
RIβ
GAPDH
Non-Reduced
RIβ dimer
75
37
140
75
D
Insoluble
Reduced
RIβ
TOPO1
Non-Reduced
RIβ monomer
75
100
140
75
E
WT
L50R
L50I
L50A
L50K
RIβ
Cα
Merge
F
Reduced
Cα WT
Cα L50R
Cα L50I
Cα L50A
Cα L50K
75
70
37
RIβ
Cα
GAPDH
G
Non-Reduced
Cα WT
Cα L50R
Cα L50I
Cα L50A
Cα L50K
140
75
60
RIβ dimer
RIβ monomer

**Supplementary Figure 8 (for main Fig. 4)** Cell lysates of transiently transfected PC12 cells expressing mKO2-tagged RIβ WT or mutants were separated by SDS-PAGE under non-reduced conditions. GAPDH was used as a loading control.

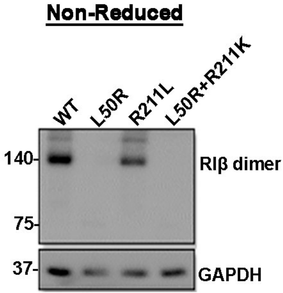
Non-Reduced
WT
L50R
R211L
L50R+R211K
140-
75-
37-
RIβ dimer
GAPDH

**Supplementary Figure 9 (for main Fig. 5) A.** Representative confocal images of PC12 cells co-transfected to express RIβ-WT: dAKAP1 or RIβ -L50R:dAKAP1 at indicated ratio. RIβ is in red, and dAKAP1 is in green. Scale bar: 20 μm. **B.** Quantification of % transfected cells with aggregates at the indicated RIβ -L50R: dAKAP1 ratios from ~150-250 cells per condition. Each dot in the graph represent % transfected cells with aggregates in an image taken at X20. One-way ANOVA with a post-hoc Dunnett's test was performed. ****$P$<0.00001, ns; non-significant comparison. **C.** Same images as in G quantified for aggregate size (μm$^2$) at the indicated RIβ -L50R: dAKAP1 ratios. One-way ANOVA with a post-hoc Tukey's HSD test was performed. *$P$<0.05, **$P$<0.01, ***$P$<0.001, ****$P$<0.0001, ns; non-significant comparison.

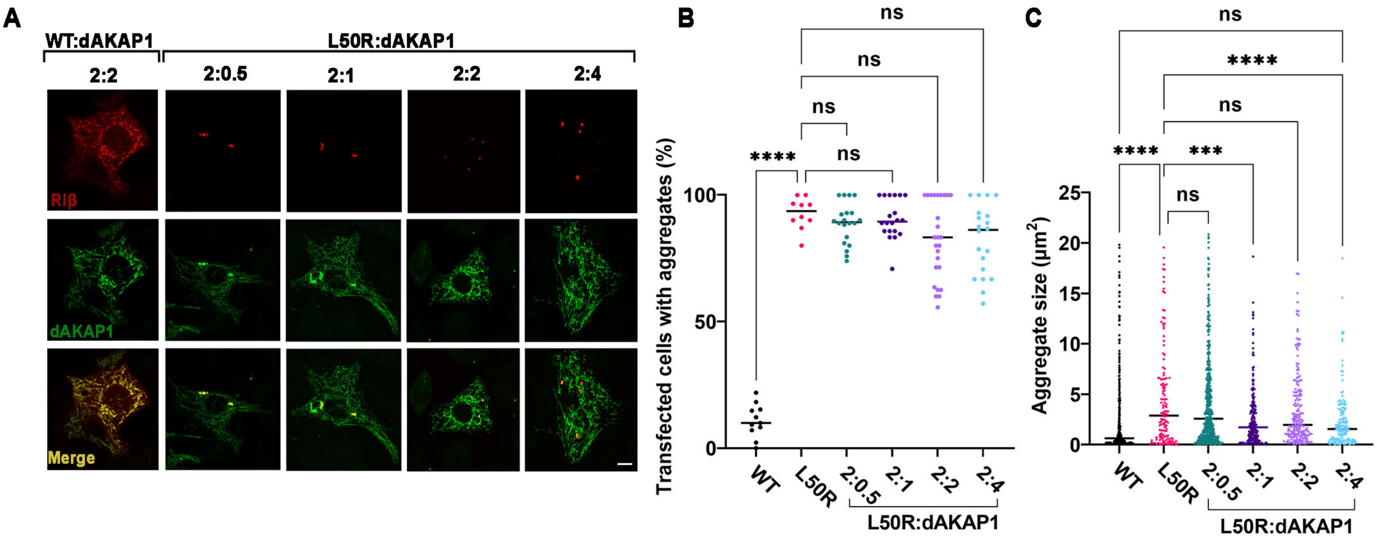

A
WT:dAKAP1
L50R:dAKAP1
2:2
2:0.5
2:1
2:2
2:4
RIβ
dAKAP1
Merge
B
ns
ns
ns
****
ns
Transfected cells with aggregates (%)
100
50
0
WT
L50R
2:0.5
2:1
2:2
2:4
L50R:dAKAP1
C
ns
****
ns
****
***
ns
Aggregate size (μm²)
25
20
15
10
5
0
WT
L50R
2:0.5
2:1
2:2
2:4
L50R:dAKAP1
