# Supplementary material for: A mutation in the PRKAR1B gene drives pathological mechanisms of neurodegeneration across species
Source: Brain. 2024 May 14;147(11):3890–905. doi: 10.1093/brain/awae154 (PMC11531844; doi:10.1093/brain/awae154)
Supplement: awae154_Supplementary_Data [file awae154_supplementary_data.zip › brain-2023-02204-File008.pdf]

Full unedited gel for Figure 2.

B1.

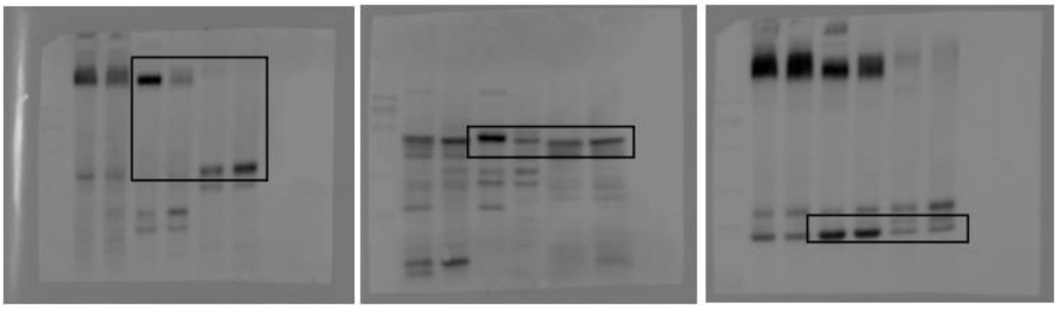

RIβ RIβ GAPDH

C1.

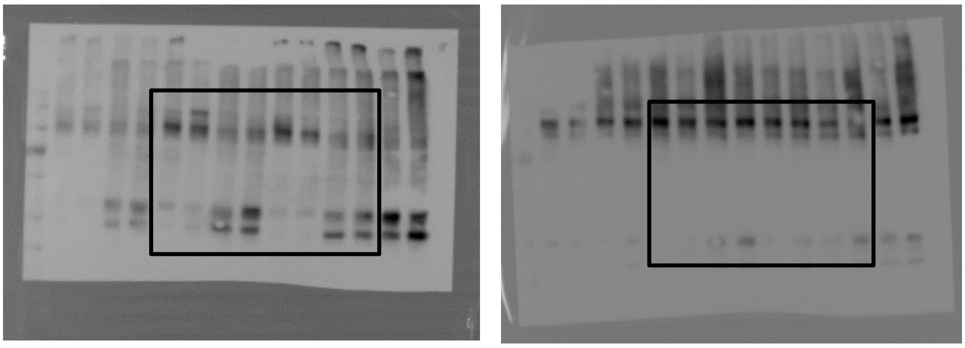

RIβ RIα

C4.

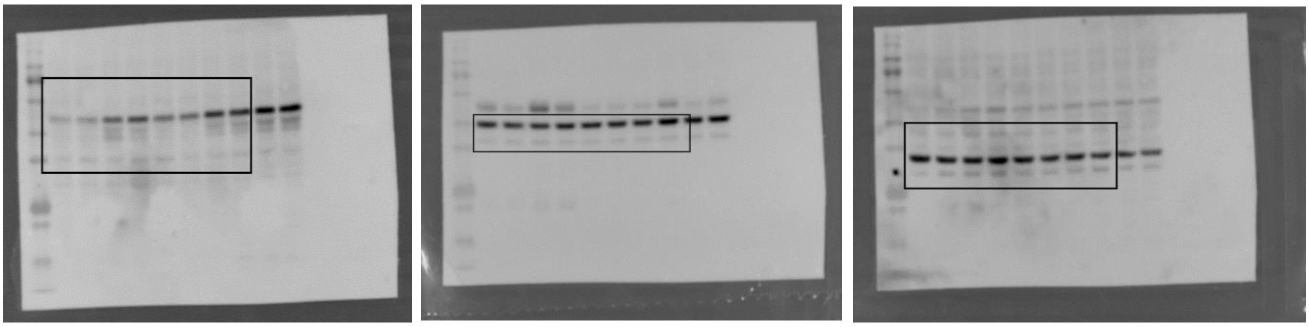

RIβ RIα GAPDH

D1.

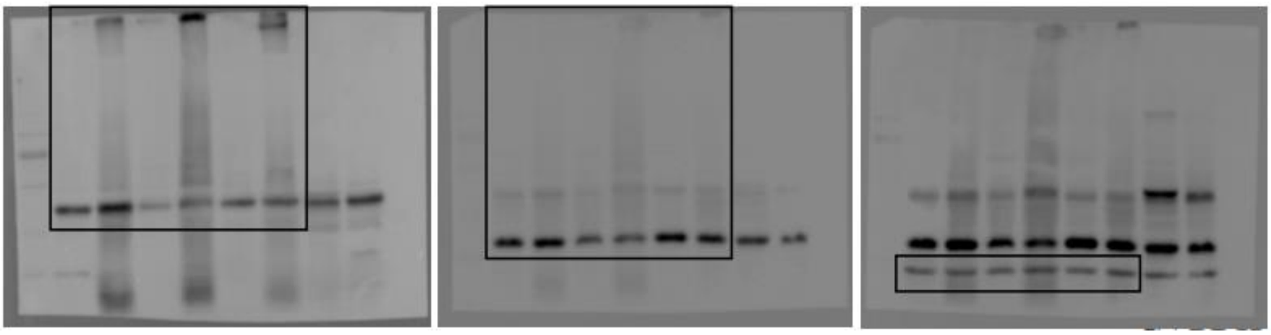

RIβ PKAC GAPDH

F1.

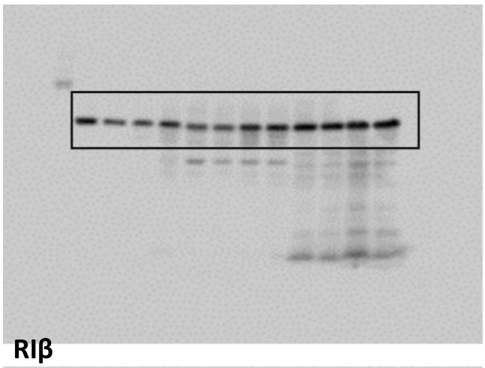

RIβ

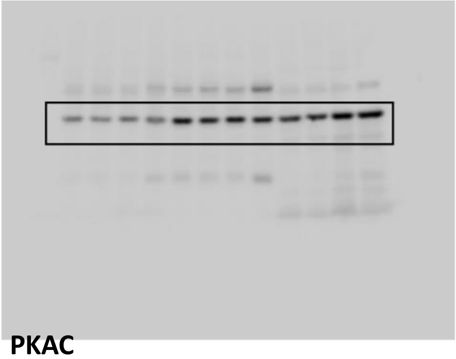

PKAC

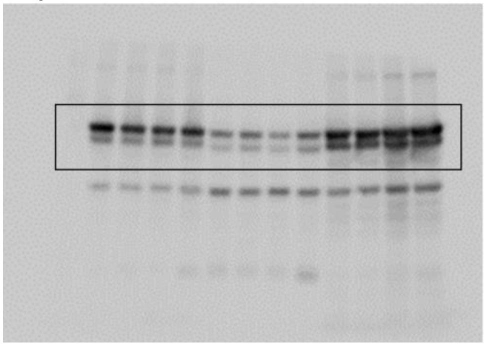

α-internexin

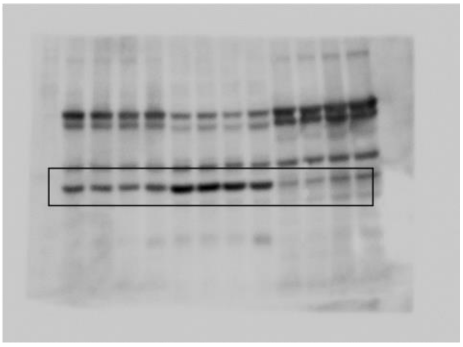

GAPDH

Full unedited gel for Figure 3.

D.

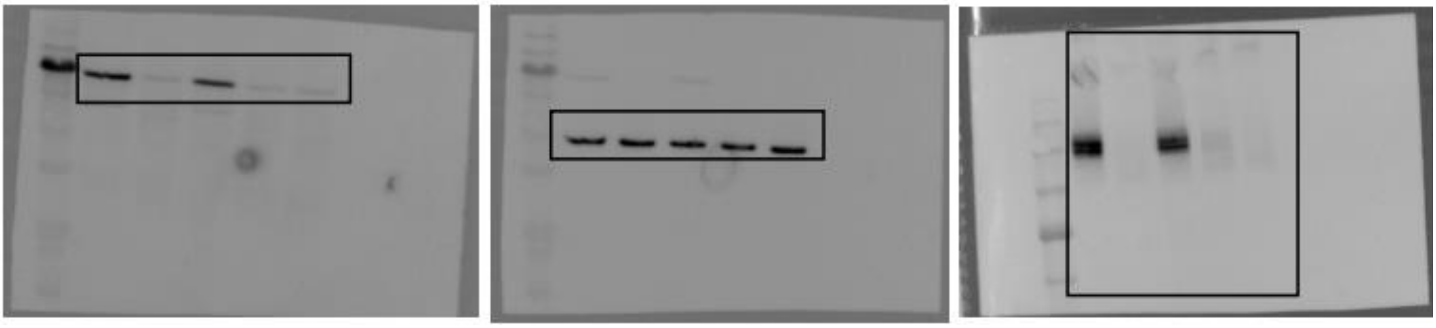

RIβ β-actin RIβ

E.

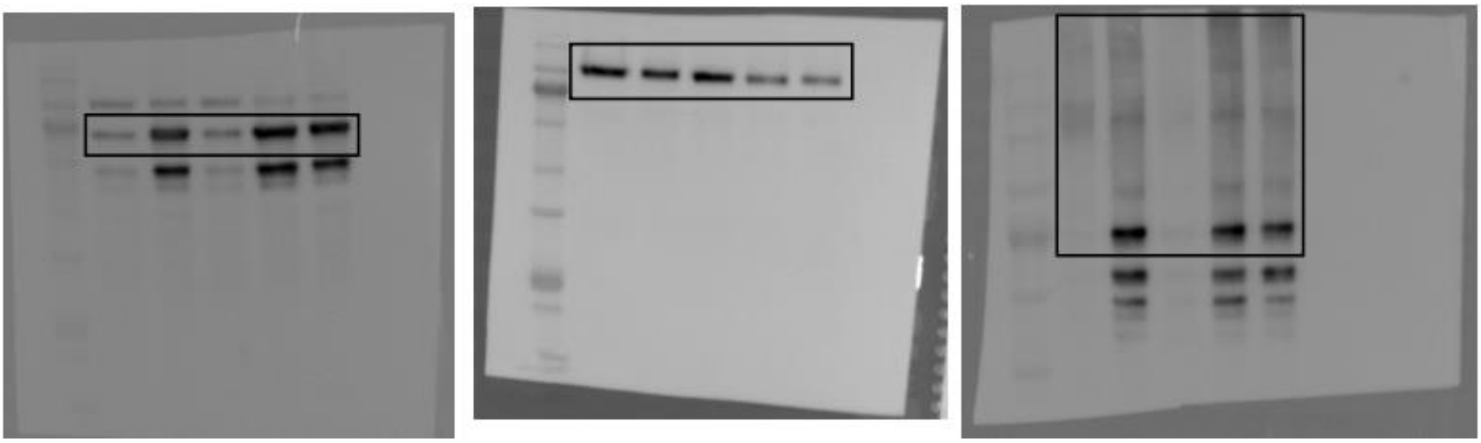

RIβ TOPO1 RIβ

G.

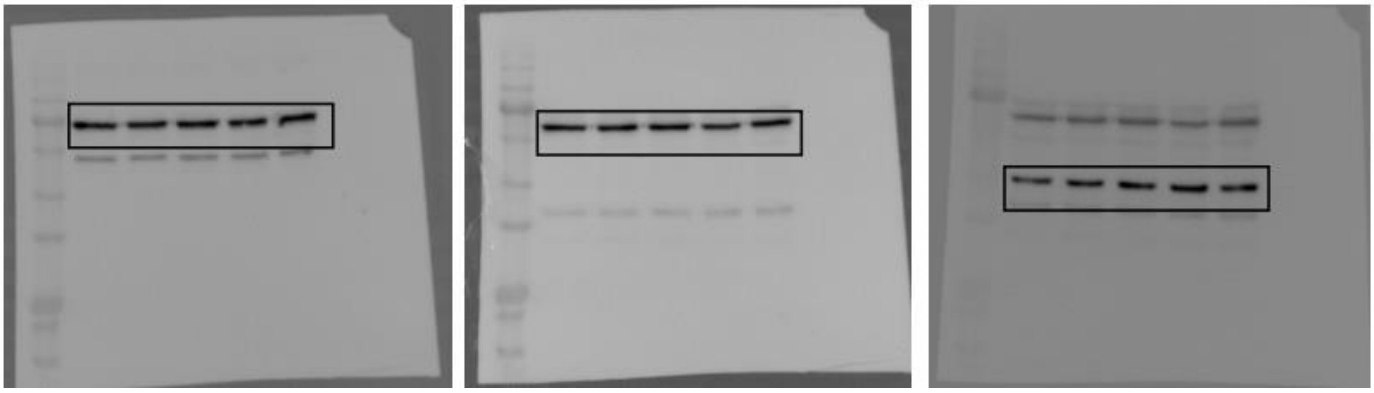

RIβ PKAC β-actin

H.

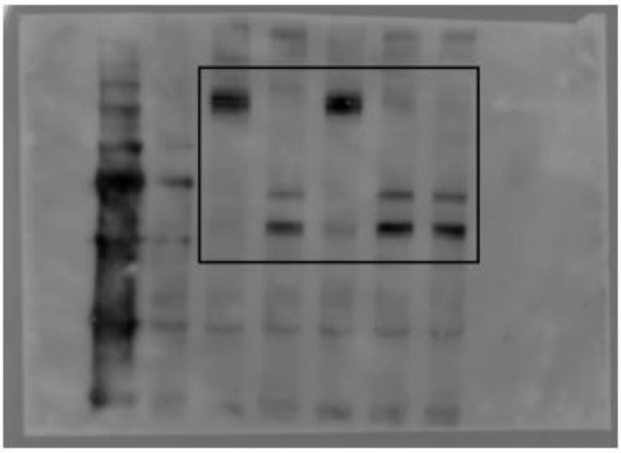

Full unedited gel for Figure 4.

E.

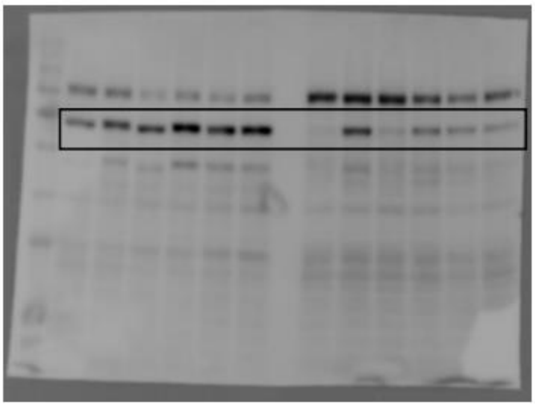

RIβ

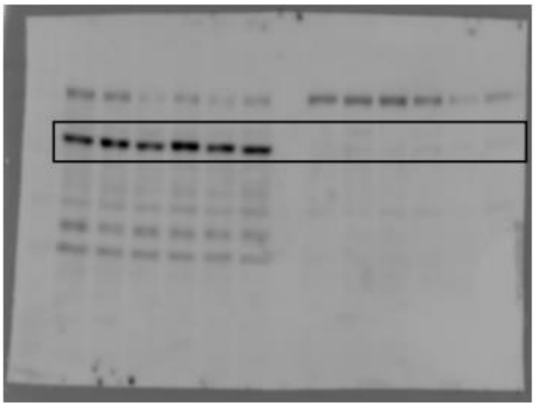

PKAC

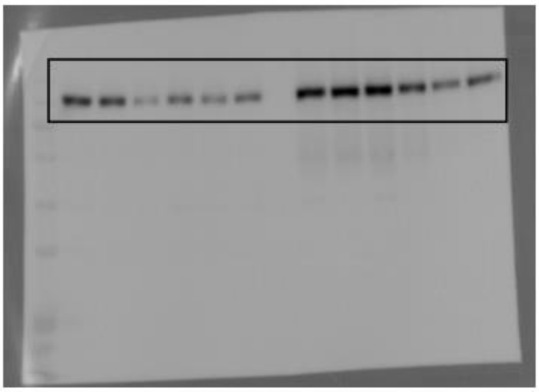

TOPO1

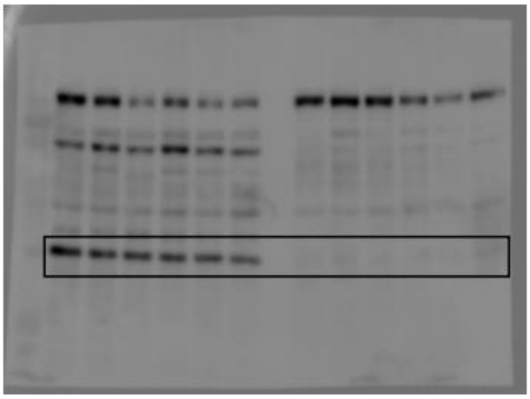

GAPDH

<u>**Full unedited gel for Figure 5.**</u>

A.

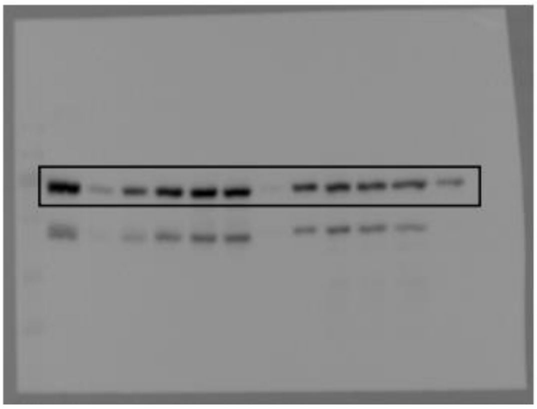

RIβ

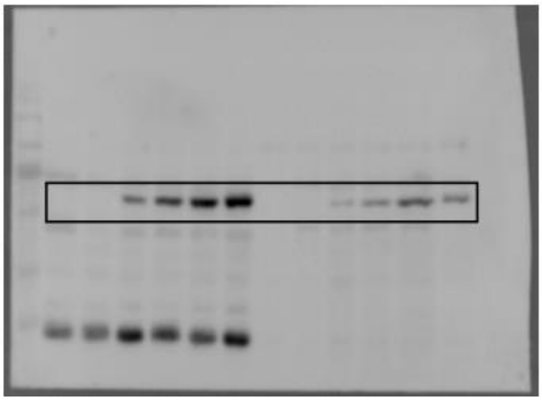

PKAC

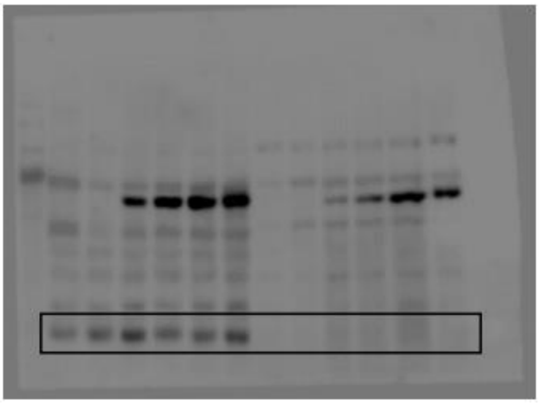

GAPDH

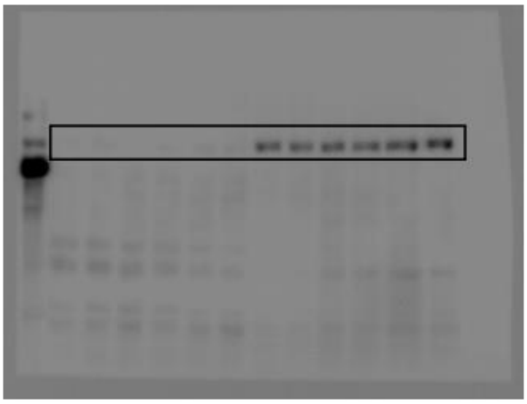

TOPO1
