# Supplementary material for: A mutation in the PRKAR1B gene drives pathological mechanisms of neurodegeneration across species
Source: Brain. 2024 May 14;147(11):3890–905. doi: 10.1093/brain/awae154 (PMC11531844; doi:10.1093/brain/awae154)
Supplement: awae154_Supplementary_Data [file awae154_supplementary_data.zip › brain-2023-02204-File009.pdf]

Supplementary Material

Extended Material and Methods

Quality control of L50R mouse model. Initially, mice were extensively back-crossed to C57BL/6J female mice (#000664, Jackson Labs) to reduce off-target mutations. Using the UCSC Genome Track based on crispor.org, we predicted 8 exonic genomic regions that may be off-target sites for the guide RNA used. We sequenced these exonic regions and no mutations were found (data not shown). In addition, the coding regions and untranslated regions (UTRs) of the RIβ gene were sequenced and no additional mutations were found. These quality control sequencing checks, along with extensive backcrossing, reduced the possibility of off-target mutations driving the phenotypes studied.

AD mouse model. An AD-Tg mouse model (B6.C3-Tg (APPswe, PSEN1dE9) 85Dbo/Mmjax, stock no. 004462) (PSAPP) and WT mice (C57BL/6J, stock no. 000664) were purchased from Jackson Laboratory (Bar Harbor, ME) and bred in-house in Dr. Rissman's lab as previously described[1,2]. The UCSD IACUC approved all experimental protocols. Male and female PSAPP mice, which contain a chimeric mouse/human *APP* gene co-expressed with a mutant human *PS1* gene were used. WT littermates were used as control. All mice were weaned at 21 days of age and entered the study at 30 days of age. Mice were housed (2 to 4 mice/cage) in a temperature controlled room (22 °C) with a 12 h light-dark cycle.

Quantitative real-time PCR (Q-PCR)

Mouse brain samples were homogenized and RNA was extracted using the mirVana Isolation Kit (ThermoFisher Scientific). Samples were resuspended in RNase-free water and quantified using

the Nanodrop. High quality RNA (200 ng) was used for cDNA preparation with the Superscript IV VILO Master Mix (ThermoFisher Scientific). Q-PCR was performed with Taqman Gene Expression Assays and Taqman Universal Mastermix (ThermoFisher Scientific). Samples were quantified in at least triplicate using Gapdh as a housekeeping gene, which was included on all plates for normalization. Q-PCR statistical analyses were performed using a 2-way ANOVA.

Behavioural Testing

Testers were blinded to genotype until analysis and mice were randomized throughout testing to reduce batch effects. All behavioural testing began at lights on (ZT0) and started with 30 minutes of acclimation to the testing room.

Elevated Zero Maze

The elevated zero maze (SD Instruments) was used to measure anxiety-like behavior in our mouse model. This test uses an elevated circular platform that has two closed areas and two open areas. The zero shape avoids the ambiguous center area seen in the elevated plus maze. Mice are not handled prior to testing. Each mouse is placed in an open area and then activity is recorded using Ethovision for five minutes. Time spent in the open area versus the closed is calculated as a measurement of anxiety-like behavior.

Rotarod Performance Test

The rotarod test was used to measure motor performance in our mouse model. This test uses an automated accelerating rod (Ugo Basile) and quantifies the motor activity of the mice by measuring the latency to fall in seconds. Timing also stops if a mouse clings to the rod and completes a full rotation. We used an increasing speed from 4-40 rpm for a maximum time of 5

minutes. Each mouse was handled for 3 minutes for 3 days prior to testing. Then, each mouse completed three trials each day with a 15 minute break for five days.

Contextual Fear Conditioning

Contextual fear conditioning was used to test learning and memory in our mouse model. This test measures the learning and memory of a context associated with an aversive stimulus (0.75mA shock). This requires a training day where the mouse explores the fear conditioning chamber for 2 minutes and 28 seconds followed by a 2 second shock. After the shock, mice remain in the chamber for 30 seconds and all movement is recorded throughout. 24 hours later, mice are tested by returning the mice to the same chamber for 5 minutes. Freezing behavior is measured using FreezeScan Version 2.0. Prior to training, mice were singly housed and handled for five days in the behavioral room by the tester.

Activity Monitoring

Activity monitoring was used to measure movement in light and dark cycles. Mice were singly housed with corn-cob bedding for one week before testing to allow acclimation to the activity chamber. Each chamber is equipped with individual fans, lights, and an infrared beam break system. Following acclimation, data was collected as activity counts (beam breaks) every 10-seconds in the horizontal direction for one week in 12-hour light:dark phase. An infrared beam-break system (Opto M3, Columbus Instruments, Columbus, OH) was used to measure locomotor activity as previously described [3]. On the first day of habituation, mice were singly housed with corncob bedding to prevent interference to the infrared beams. Food was placed at

the bottom of the cages along with water bottles. Habituation occurred for 7 days before data collection, which was collected for a further 7 days.

Three-chamber Social Preference Test.

The social preference test measures the mouse model's preference between a social stimulus or a novel object as described earlier[4]. Mice were placed in a black area divided into three compartments with a wide opening between compartments. In each outer compartment, a clear acrylic perforated cylinder was placed in the center. Mice were habituated to the arena for 10 minutes before testing. Then, a novel same sex gonadectomized mouse was placed in one cylinder and a novel, colored plastic block was places in the other cylinder. The mice were placed in the middle compartment and activity was tracked by EthoVision software for distance traveled, velocity and interaction time (calculated as previously described[4]). Mice were excluded from analyses if they climbed to the tops of the walls of the arena.

Statistical Analysis.

All statistical analyses for behavioral tests were completed in GraphPad Prism. Data generated from the elevated zero maze was compared to determine the percentage in the open arm (seconds in the open arm/ seconds in open and closed arm). Total distance moved was also calculated and analyzed using the Student's t-test. Data generated from the rotarod test was analyzed using a 2-way repeated measures ANOVA with multiple comparisons for each trial. Data generated from the fear conditioning tests compared percentage freezing during the training before the shock (baseline) to the percentage freezing during the testing. A 2-way repeated measures ANOVA was used to determine significance and a P-value less than 0.05 was

considered significant. Results were expressed as means +/- SEM. Values of $P < 0.05$ were considered as statistically significant.

Plasmids and PCR-site directed mutagenesis

The mKO2-tagged PKA regulatory subunit-expressing plasmid (mKO2-RIβ) was created as described earlier [5]. The mCerulean-tagged PKA C-subunit-encoding plasmid (mCerulean-PKA-Cα) and the mCerulean-tagged AKAP-encoding plasmid (mCerulean-AKAP), were kindly provided by the Susan Taylor lab (University of California, San Diego, CA). Single-site mutations (i.e., mutations that lead to the replacement of leucine at position 50 of the RIβ to an arginine, isoleucine, alanine or lysine) were introduced into the mKO2-RIβ template using a site-directed mutagenesis kit (New England BioLabs, Ipswich, MA). The mKO2-RIβ (L50R) plasmid was used as a template to replace the arginine at position 211 of RIβ to a lysine. All constructs were sequenced to confirm the presence of the desired mutation.

Antibodies

Primary antibodies: Sheep anti-PKA RIβ antibodies (R&D systems catalog # AF4177 (RRID: AB_2284184)) diluted 1:2,500 for Western Blot, 1:100 for immunohistochemistry (IHC). Mouse anti-PKAC Monoclonal Antibody BD Biosciences catalog # 610981 (RRID: AB_398294) dilution 1:4,000 for WB. Rabbit anti-Human PRKACB LSBio catalog # LS-C191947-100 dilution 1:100 for IHC. Rabbit anti-GAPDH Abcam Catalog # ab9485 (RRID: AB_307275) dilution 1:2,500 for WB. Rabbit anti α-internexin Abcam Catalog # ab40758 (RRID:AB_726960) dilution 1:2,500 for WB. Rabbit anti-Calbindin Abcam Catalog # ab108404 (RRID:AB_10861236) dilution 1:500 for IHC, Anti-Topoisomerase I Abcam Catalog # ab109374 (RRID: AB_10861978) dilution 1:1,000 for WB. Anti-beta Actin Abcam Catalog #

ab119716 (RRID: AB_10898702) dilution 1:5,000 for Western blots (WB). The WB primary antibodies were prepared in 1% filtered BSA in PBST solution, and the IHC primary antibodies were prepared in 1:10 blocking solution (10% Normal Goat Serum (RRID: AB_2336990) + 0.1% filtered BSA) in PBST.

Secondary antibodies: Donkey Anti-Sheep IgG, HRP Conjugated (Abcam) (RRID: AB_955452), Goat Anti-Rabbit IgG, HRP Conjugated (Abcam) (RRID: AB_955447), Rabbit to Mouse IgG, HRP Conjugated (Abcam) (RRID: AB_955440), Donkey Anti-Sheep IgG (Alexa Fluor 647) Abcam Catalog # ab150179, Goat Anti-Rabbit IgG (L+H) (Alexa Fluor 488) Invitrogen Catalog # A-11934. All HRP-secondary antibodies were used at 1:10,000 dilution and all fluorescent-secondary antibodies were used at 1:250 dilution. Rabbit anti-Sheep IgG, Biotin Conjugated (Abcam) (RRID: AB_954917), Goat anti-Rabbit IgG, Biotin Conjugated (Abcam) (RRID:AB_954902), All biotin-secondary antibodies were used at 1:100 dilution.

Transient transfection

Cells ($3X10^5$) were seeded on 6-well culture plates until a confluency of approximately 70% was reached. The following day, cells were transfected with polyethyleneimine (PEI) transfection agent (Sigma 408727). Transfected cells expressing 3μg of native or mutant RIb were transfected to express mCerulean-PKA-Cα or mCeruleanAKAP at 1:1 ratio or not, as indicated in the figures. Plasmids were mixed with 3μg PEI according to the manufacturer's instructions. Transient transfection was also performed with the calcium phosphate method to confirm consistency of the results. In this method, seeded cells were transfected with 5μg plasmids. The next day, cells were washed with DMEM high glucose medium. 48 hours after transfection, the cells were harvested for Western blot analysis or fixed for IHC.

Biochemical fractionation of cells and tissues

Human lysates: Flash-frozen postmortem human brain samples from healthy individuals (RIβ L50) or affected patients (RIβ L50R) were extracted following dissolving of equal amounts (10mg) into a buffer (TBSx1, 50mM Tris-HCl pH 7.4, 150mM NaCl, 2mM EDTA, 1% Triton X-100, 1% NP-40, 0.1% SDS, 10mM NaF, 1mM Na_3VO_4, a cocktail of protease and phosphatase inhibitors (1:100)). The lysates were homogenized using a Dounce homogenizer and sonicated 3X(10sec pulses of 30% amplitude). Equal amounts of proteins were separated by SDS-PAGE under reduced conditions. This extraction is referred as total protein.

To isolate soluble and insoluble fractions from flash-frozen post-mortem human brain samples from healthy individuals (RIβ L50) and affected patients (RIβ L50R), samples were weighed (100mg) and dissolved in TBSx1 including a cocktail of protease and phosphatase inhibitors (concentrations 1:100). The samples were homogenized using a Dounce homogenizer. The lysates were ultracentrifuged (4°C,1h, 100000g) centrifuged in benchtop refrigerated centrifuge (4°C, 45min, 15000 rpm). The supernatant was called the soluble fraction. The pellet was washed three times with TBSx1 and then dissolved with SDS buffer (TBSx1+ 5% SDS + inhibitors). The insoluble fraction was sonicated 3X (10sec pulses of 30% amplitude).

Mouse lysates: Different mouse brain regions were suspended in 300µl TBSX1 with a cocktail of protease inhibitors and phosphatase inhibitors (Sigma P8340; 1:100) and homogenized on ice. 100µl of each sample was transferred as 'total protein' and suspended with 100µl RIPA lysis buffer (50mM Tris pH 7.4, 150mM NaCl, 2mM EDTA, 1%TritonX-100, 1% NP-40, 0.1% SDS, 1 mM DTT, protease and phosphatase inhibitors (1:100)). The remaining 200 µl were centrifuged at 21,130g for 90 minutes. The supernatants were keeped as soluble fraction and the resulting pellet was resuspended three times in TBS, centrifuged at 21,130g for 45 min

and suspended in 200µl 5% SDS and 8M UREA that added to TBS with phosphatase and protease (insoluble fraction). Total protein and the insoluble fraction were sonicated.

Cell lysates: Following transfection, cells were washed in cold PBS twice, and then harvested with a cold lysis buffer (50mM Tris-HCl pH7.4, 150mM NaCl, 1mM EDTA, 0.1% Triton X-100, 1% NP-40, 10mM NaF, 1mM Na_3VO_4, cocktail of protease inhibitors and phosphatase inhibitors (Sigma P8340) (1:100). Cell lysates were kept on ice for 1 hour and subjected to centrifugation for 45 min at 4ºC and 15000 rpm. The supernatant was collected and termed the soluble fraction. Following several washes, the pellet was resuspended with 5% SDS and 6M urea in lysis buffer and sonicated for three cycles (10sec pulses of 30% amplitude) and termed the insoluble fraction. Soluble and insoluble fractions were resuspended in Laemmli sample buffer (4% SDS, 20%glycerol, 0.004% bromphenol blue, 0.125M Tris-Cl, pH6.8, 10% 2-mercamtoethanol) boiled and separated by SDS-PAGE. For dimer analysis, samples were resuspended in Laemmli buffer without a reducing agent and SDS and were not heated.

SDS-PAGE and Western blot

Proteins concentrations were measured by Bradford assay in a plate reader (i-CONTROL). 25-50µg of each protein sample were mixed with 5x sample buffer (with or without a reducing agent and SDS) and resolved by 7% or 10% SDS-PAGE. The reduced samples were heated at 95ºC for 5 min before loading on gels. The non-reducing samples were not boiled. The gels were transferred to PVDF membranes (Bio-Rad). The membranes were blocked with 5% bovine serum albumin (BSA)(Sigma) in PBST for 1 hour at room temperature. Membranes were incubated overnight at 4˚C with indicated primary antibodies. Following 3X washes with PBST, membranes were incubated with secondary antibodies conjugated with HRP

for 1 hour. Following 3X washes with PBST, membranes were incubated with ECL reagent. Signals were visualized with the enhanced chemiluminescence system UVITEC CAMBRIDGE.

Immunohistochemistry staining and imaging

Human samples:

DAB staining: Paraffin-embedded human brain tissues were heated in an incubator at 60°C for 40 min. Following tissue de-paraffinizion with xylene and rehydration in a graded series of increasing concentrations of alcohol, the samples were treated with peroxidase blocking solution (3% H_2O_2 in methanol) for 20 min at room temperature. Antigens were retrieved by boiling with sodium citrate buffer, pH 6. The tissues were then blocked using 10% normal goat serum in PBST and 0.1% BSA for 1 h at room temperature. Primary antibodies were incubated overnight at 4°C. The next day, the slides were washed and incubated with biotinylated secondary antibodies for 2 hours at room temperature. Tissues were incubated with ABC solution (Vector Laboratories) for 1 hour at room temperature. Following three washes with PBST for 5 min each, the tissues were stained with DAB solution (Vector Laboratories) for 10 min. Tissues were incubated with hematoxylin (Abcam) for nucleus staining. The slides were covered with Gelvatol and viewed with Zeiss 506 color camera (14 bit) in an Upright Apotome microscope (Zeiss). The images were stitched together to a larger picture to obtain a full view of the tissue. The images were acquired with Zen-Black software (2011).

Immunofluorescence staining: Paraffin-embedded human brain tissues were de-paraffinized with xylene and rehydrated in a graded series of increasing concentrations of alcohol and water. Antigens were retrieved by boiling with sodium citrate buffer, pH 6. The tissues were then blocked using 10% normal donkey serum in PBST and 0.1% BSA for 1 hour at room

temperature. Primary antibodies were incubated overnight at 4ºC. On the next day, the slides were washed and incubated with fluorescence secondary antibodies for 2 hours at room temperature. The tissues were stained with TrueBlack Lipofuscin Autofluorescence Quencher (Biotium) for 1 minute. To stain the nuclei, the cells were incubated for 5 min with 0.05 µg/ml Hoechst solution (Invitrogen). The slides were covered with Gelvatol and photographed under a Upright Apotome microscope (Zeiss). The images were stitched together to form a larger picture to obtain a full view of the tissue. The images were acquired with Zen-Black software (2011).

Mouse brains:

Mouse brains were sectioned with Vibratome (Leica VT 1200s) in 80µm and transferred to Cryopotectant solution (30% glycerol, 30% ethylene glycol in PBS) and stored at -20ºC. The tissue slices were selected and washed five times in PBS, kept permeabilized in PBS containing 0.2% Triton X-100 at room temperature for 20 min. Then 4 washes with buffer containing PBS and 50mM Glycine following blocking (5% NGD and 1% BSA in PBS with 0.1% TritonX-100) for 1 hour and probed with primary antibodies at 4ºC for overnight. Then, 4 washes with working buffer (blocking with PBS with 0.1% Triton X-100 (1:9)) were completed and the samples probed with secondary antibody for 2 hours, followed by 2 washes with PBS, which was then applied for detection. To stain the nuclei, the cells were incubated for 5 min with 0.05µg/ml Hoechst solution (Invitrogen). The brain tissues were examined and photographed under a confocal laser-scanning microscope (Leica STED Live Imaging) and Upright Apotome microscope (Zeiss).

Cell culture:

Approximately 48 hours after transient transfection or co-transfection, the coverslips were rinsed twice with phosphate buffered saline (PBS) (Biological Industries) and fixed with 4% paraformaldehyde (Electron Microscopy Science) for 15 min. Coverslips were stained with Hoechst stain (dilution 1:10000) (Invitrogen) for 5 min and mounted on cover glasses with Gelvatol. Fixed cells were imaged with a confocal laser scanning microscope (Zeiss LSM 780). Confocal images were acquired with Zen- Black software (2011).

Live imaging experiment

PC12 cells were seeded on a 35 mm glass bottom dish (300,000 cells/well; Cellivis) and allowed to adhere to the cover glass bottom overnight. The following day, the cells were co-transfected with indicated plasmids using the calcium phosphate method. After 24 h, the cells were washed with fresh medium. Forty-eight h post-transfection, the medium was removed and replaced with free medium (without fetal bovine serum). The cells were then pre-warmed (37ºC) for 1 h and imaged under a confocal microscope with a stage-top incubation chamber with 5% CO_2 (Ziess LSM780 inverted confocal microscope). Five positions were selected for imaging from each slide. Before treatment, the cells were imaged for 25 min (5 min each cycle). The cells were treated with 20 μM FSK (ABCAM) and 200 μM IBMX (Sigma) and imaged over the next 45 min (9 cycles od 5 min each). Following treatment, the cells were washed with fresh DMEM including 10% FBS three times and imaged for 130 min. Images were processed using image J software.

Quantification

A customized code written in Java for the Fiji (Version 1.53r 21 April 2022) Image processing platform[6] was used to quantify aggregate numbers and sizes. Images taken at X20 magnification were treated as follows:

Quantification for human brain images:

Pixel intensity normalization was achieved by multiplying each pixel with a fixed factor of 24.5. The factor was calculated based on a reference WT image, so that at the end 0.0005% of the reference image's pixels were saturated. The image was converted to a binary image using the 'Default' B&W algorithm with a fixed intensity threshold of 45,000. The fixed threshold was chosen based on intensity values derived from native images and after noticing that less than 0.01% of the pixels had that high intensity level. Every image was marked with 100 distinct rectangles each 200.59 µm X 200.59 µm in size. Fiji's particle analysis was run for each of the rectangles, counting the number of aggregates and their size.

Quantification for mouse brain images:

Samples were run through a median filter with radius=1 (pixels) to smooth the image and thereby reduce the impact of neglectable small artifacts, i.e., one-pixel sized areas with high intensity due to unrelated reasons. Background subtraction was implemented with a rolling ball radius=500 (pixels). Pixel intensity normalization was achieved by multiplying each pixel with a factor so that at the end 0.0012% of the image's pixels were saturated. The image was converted to a binary image using the 'Default' B&W algorithm with a fixed intensity threshold of 45,000. The fixed threshold was chosen based on intensity values derived from native images and after noticing that less than 0.01% of the pixels had that high intensity level. We ran a particle analysis, calculating the number of aggregates and their size.

Quantification for Cell culture images:

Samples were run through a median filter with radius=1 (pixels) to smooth the image and thereby reduce the impact of neglectable small artifacts, i.e., one-pixel sized areas with high intensity due to unrelated reasons. Background subtraction was implemented with a rolling ball radius=20 (pixels). The image was converted to a binary image using the 'Default' B&W algorithm with a fixed intensity threshold of 45,000. The fixed threshold was chosen based on intensity values derived from native images and after noticing that less than 0.01% of the pixels had that high intensity level. We ran a particle analysis, calculating the area of each aggregate, and removed aggregates with areas greater than a threshold of 20 μm^2. These large aggregates occur due to hyper-transfection of plasmids encoding the measured proteins and death of the cell. It is important to note that the quantification performed was by no means an absolute but rather relative.

Co-Immunoprecipitation (Co-IP)

Mouse brains were perfused and stored at -80ºC. Extracted protein was examined by SDS-PAGE and Western Blot, as described in the SDS-PAGE and Western Blot (input) sections. Several brain regions were homogenized in NP-40 lysis buffer (1% NP-40, 50mM Tris pH 8.0, 150mM NaCl, 1mM PMSF). Protein extract was incubated overnight with a primary antibody and then the mixture was incubated with 50 µl Protein G Sepharose 4 Fast Flow beads (Cytiva) for one hour. After three washes with NP-40 lysis buffer and 1 wash with wash buffer (50mM Tris pH 8.0), the mixture was heated in SBX1, and the supernatant was loaded on SDS-PAGE followed by Western Blot analysis.

Renilla luciferase protein complimentary assay (PCA)

The Rluc PCA-based hybrid proteins PKAC-F[2] and PKI-F[2] were designed as previously described[7,8]. Rluc PCA fusions of RIβ and smAKAP were generated using an analogous cloning approach. Following PCR amplification of the RIβ and smAKAP genes, either -F[1] or -F[2] of the Rluc PCA we 5'-fused. We sub-cloned the PCR products into the 5`end of the DNA encoding the 10 amino acid-long linker $(GGGGS)_2$ and the Rluc PCA fragments (pcDNA3.1 backbone vector). We generated the RIβ[L50R] mutant using site-directed mutagenesis. For Rluc PCA analyses, HEK293 cells were grown in DMEM supplemented with 10% fetal bovine serim. We transiently over-expressed the indicated versions of the Rluc-PCA-based reporter constructs in a 24-well plate format. Twenty-four or 48 h post-transfection, we exchanged the growth medium and resuspended cells in PBS. Cell suspensions were transferred to 96-well plates and subjected to bioluminescence analyses using the LMaxTMII-384 luminometer (Molecular Devices). Rluc bioluminescence signals were integrated for 10 seconds following addition of the Rluc substrate benzyl-coelenterazine (5 µM; Nanolight).

References

1. Zhang, C., Kuo, C.C., Moghadam, S.H., Monte, L., Campbell, S.N., Rice, K.C., Sawchenko, P.E., Masliah, E., and Rissman, R.A. (2016). Corticotropin-releasing factor receptor-1 antagonism mitigates beta amyloid pathology and cognitive and synaptic deficits in a mouse model of Alzheimer's disease. Alzheimer's and Dementia *12*, 527–537. 10.1016/j.jalz.2015.09.007.
2. Campbell, S.N., Zhang, C., Monte, L., Roe, A.D., Rice, K.C., Taché, Y., Masliah, E., and Rissman, R.A. (2015). Increased tau phosphorylation and aggregation in the hippocampus of mice overexpressing corticotropin-releasing factor. Journal of Alzheimer's Disease *43*, 967–976. 10.3233/JAD-141281.
3. Angelakos, C.C., Watson, A.J., O'Brien, W.T., Krainock, K.S., Nickl-Jockschat, T., and Abel, T. (2017). Hyperactivity and male-specific sleep deficits in the 16p11.2 deletion mouse model of autism. Autism Research *10*, 572–584. 10.1002/AUR.1707.

4. Lauffer, M., Wen, H., Myers, B., Plumb, A., Parker, K., and Williams, A. (2022). Deletion of the voltage-gated calcium channel, CaV1.3, causes deficits in motor performance and associative learning. Genes Brain Behav *21*. 10.1111/gbb.12791.

5. Ilouz, R., Lev-Ram, V., Bushong, E.A., Stiles, T.L., Friedmann-Morvinski, D., Douglas, C., Goldberg, G., Ellisman, M.H., and Taylor, S.S. (2017). Isoform-specific subcellular localization and function of protein kinase A identified by mosaic imaging of mouse brain. Elife *6*. 10.7554/eLife.17681.

6. Schindelin, J., Arganda-Carreras, I., Frise, E., Kaynig, V., Longair, M., Pietzsch, T., Preibisch, S., Rueden, C., Saalfeld, S., Schmid, B., et al. (2012). Fiji: an open-source platform for biological-image analysis. Nat Methods *9*, 676–682. 10.1038/NMETH.2019.

7. Rock, R., Mayrhofer, J.E., Bachmann, V., and Stefan, E. (2015). Impact of kinase activating and inactivating patient mutations on binary PKA interactions. Front Pharmacol *6*, 170. 10.3389/fphar.2015.00170.

8. Stefan, E., Aquin, S., Berger, N., Landry, C.R., Nyfeler, B., Bouvier, M., and Michnick, S.W. (2007). Quantification of dynamic protein complexes using Renilla luciferase fragment complementation applied to protein kinase A activities in vivo. Proc Natl Acad Sci U S A *104*, 16916–16921. 10.1073/pnas.0704257104.
